# Secondary Metabolites from the Coral-Derived Fungus *Aspergillus austwickii* SCSIO41227 with Pancreatic Lipase and Neuraminidase Inhibitory Activities

**DOI:** 10.3390/md21110567

**Published:** 2023-10-29

**Authors:** Ying Chen, Yanchun He, Xiaoyan Pang, Xuefeng Zhou, Yonghong Liu, Bin Yang

**Affiliations:** 1 CAS Key Laboratory of Tropical Marine Bio-Resources and Ecology/Guangdong Key Laboratory of Marine Materia Medica, South China Sea Institute of Oceanology, Chinese Academy of Sciences, Guangzhou 510301, China; chenying21@mails.ucas.ac.cn (Y.C.); heyanchun22@mails.ucas.ac.cn (Y.H.); xypang@scsio.ac.cn (X.P.); xfzhou@scsio.ac.cn (X.Z.); 2 University of Chinese Academy of Sciences, 19 Yuquan Road, Beijing 100049, China

**Keywords:** *Aspergillus austwickii* SCSIO41227, secondary metabolites, pancreatic lipase, neuraminidase, molecular docking

## Abstract

The coral-derived fungus *Aspergillus austwickii* SCSIO41227 from Beibu Gulf yielded four previously uncharacterized compounds, namely asperpentenones B–E (**1**–**4**), along with twelve known compounds (**5**–**16**). Their structures were elucidated using HRESIMS and NMR (^1^H and ^13^C NMR, HSQC, HMBC), among which the stereo-structure of compounds **1**–**3** was determined by calculated ECD. Furthermore, compounds **1**–**16** were evaluated in terms of their enzyme (acetylcholinesterase (AChE), pancreatic lipase (PL), and neuraminidase (NA)) inhibitory activities. These bioassay results revealed that compounds **2** and **14** exerted noticeable NA inhibitory effects, with IC_50_ values of 31.28 and 73.64 μM, respectively. In addition, compound **3** exhibited a weak inhibitory effect against PL. Furthermore, these compounds showed the potential of inhibiting enzymes in silico docking analysis to demonstrate the interactions between compounds and proteins.

## 1. Introduction

The coral-derived fungi, characterized by their easy cultivation and high productivity, have been demonstrated to be an essential source region for marine natural compounds [1]. Numerous novel secondary metabolites with remarkable bioactivities, including antitumor, antibacterial, antiviral, anti-inflammatory, and other biological activities, have been discovered in coral-associated fungi [2,3,4]. The fungal genus *Aspergillus* is a highly significant group of microorganisms derived from coral, encompassing more than 180 species capable of producing bioactive compounds such as terpenoids, polyketides, alkaloids, sterols, peptides, and others [5]. Furthermore, *Aspergillus* has been implicated in the pathogenesis of various diseases affecting a wide range of organisms. An example is the occurrence of aspergillosis in sea fan corals which has resulted in mortality rates ranging from 20% to 90% in the Florida Keys [6]. Over thirty compounds have been documented from *Aspergillus sydowii* in the scientific literature, exhibiting diverse biological activities, such as phytocidal, antimicrobial, antiviral, and cytotoxic activity [7]. Their potential uses in medicine, agriculture, and other fields are vast and could lead to the development of new therapeutic agents, disinfectants, and pesticides. 

The pancreatic lipase enzyme plays a pivotal role in the metabolism of dietary fat and impedes its absorption through the small intestine, thereby exerting a direct impact on the underlying cause of obesity. The inhibition of PL directly influences the root cause of obesity [8]. Obesity, which has become an epidemic in many parts of the world, is a complex health issue that affects millions of people. Hence, the investigation of pancreatic lipase inhibitors can reveal new agents that would help combat obesity. Additionally, neuraminidases are implicated in the pathogenesis of various diseases. However, it is important to note that the use of NA inhibitors for the treatment of pancreatic cancer is still in its early stages. NA inhibitors have been associated with the potential treatment of chemotherapy-resistant pancreatic cancer [9]. More research is needed to understand their full potential and the possible side effects they may cause.

**Figure 1:**
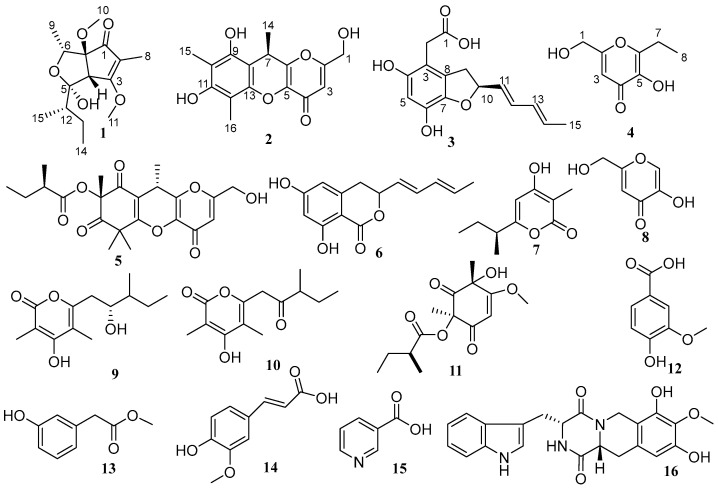
Chemical structures of **1**–**16**.

During our continued efforts to discover structurally novel and biologically active secondary metabolites from coral-derived fungi, we successfully isolated two unprecedented polyketides, namely asperpentenones B (**1**) and C (**2**), one new asperfuran named asperpentenone D (**3**), and a previously unreported kojic acid dipalmitate known as asperpentenone E (**4**) (Figure 1). In addition, twelve known compounds were also identified in the extract of *Aspergillus austwickii* SCSIO41227 obtained from the Beibu Gulf coral. The chemical diversity of these metabolites encompassed polyketides, alkaloids, and benzene derivatives. Notably, compound **1** displayed a rare cyclopentenone–tetrahydrofuran moiety. Herein, we present the comprehensive isolation process, structural elucidation results, and bioassay screening outcomes of all the isolated compounds. Our findings contribute to the understanding of these compounds and pave the way for their further exploration and utilization in a wide range of industries.

## 2. Results and Discussion

**Figure 2:**
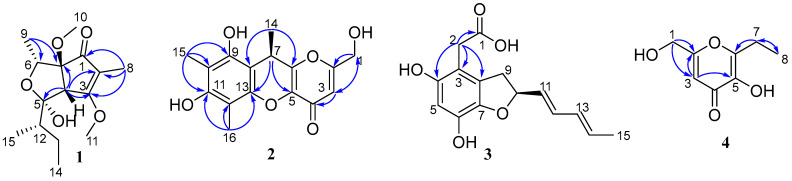
Key HMBC correlations of compounds **1**–**4**.

**Figure 3:**
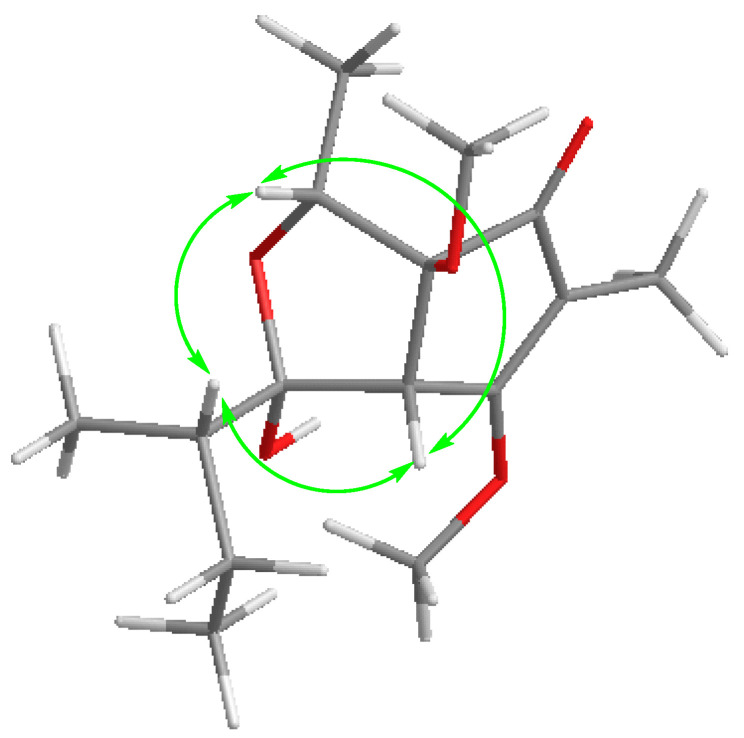
Key NOESY correlations of compound **1**.

**Figure 4:**
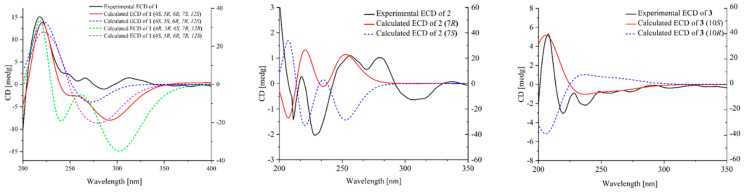
Experimental and calculated ECD spectra of compounds **1**–**3**.

Compound **1** was obtained as a brown oil with a molecular formula of C_15_H_24_O_5_, determined via HRESIMS analysis (*m*/*z* 285.1698 [M + H]^+^) (Figure S7). A comprehensive analysis of its ^1^H NMR data in DMSO-*d*_6_ revealed the presence of three methines (*δ*_H_ 3.99 (q, *J* = 6.4 Hz, H-6); 3.56, (d, *J* = 1.7 Hz, H-4); and 1.66, (m, H-12)); four methyls (*δ*_H_ 1.68, (d, *J* = 1.5 Hz, 8-CH_3_); 0.89, (t, *J* = 7.4 Hz, 9-CH_3_); 1.10, (d, *J* = 6.5 Hz, 15-CH_3_); and 0.96, (d, *J* = 6.7 Hz, 14-CH_3_)), and two methoxy moieties (*δ*_H_ 4.06 (s, 3-OCH_3_) and 3.04 (s, 7-OCH_3_)). The analysis of the ^13^C NMR data, employing distortionless enhancement via polarization transfer (DEPT) and heteronuclear singular quantum coherence (HSQC) spectra analysis, revealed the presence of fifteen carbon signals. These included four methyls (*δ*_C_ 7.0 (C-8), 12.4 (C-9), 13.0 (C-15), and 13.8 (C-14)), two methoxy groups (*δ*_C_ 51.3 (C-10) and 58.9 (C-11)), and one carbonyl (*δ*_C_ 200.6 (C-1)). The planar structure of **1** was elucidated through correlation spectroscopy (COSY) and heteronuclear multiple bond connectivity (HMBC) spectra analysis. The presence of a cyclopentenone ring containing an α,β-unsaturated motif (*δ*_C_ 200.6 (C-1), 113.0 (C-2), 181.2 (C-3), 53.5 (C-4), and 91.7 (C-7)) was confirmed through HMBC correlations between H-4 and C-2/C-3/C-7, and between H3-8 and C-1/C-2/C-3 (Figure 2). In addition, the identification of two methoxy groups located at C-3 and C-7 on the cyclopentenone ring was achieved by analyzing HMBC correlations involving H3-11 and H3-10, respectively. The NMR spectroscopic data of **1** (Table 1, Figures S1–S5) closely resembled that of the known compound asperpentenone A [10], with the sole distinction being the absence of a methyl group at C-5, which is consistent with the molecular formula. The structure of compound **1**, along with its relative configuration, was confirmed through HMBC and NOESY spectra (Figure 2 and Figure 3). The NOESY correlations indicate that these protons of H-4 (*δ*_H_ 3.56), H-6 (*δ*_H_ 3.99), and H-12 (*δ*_H_ 1.66) are located on the same face of the ring system (Figure 3). The absolute configuration of **1** was further confirmed by comparing the calculated results with experimental ECD and the results reported in the literature (Figure 4 and Figure S6). By integrating the pertinent information obtained from NOESY spectra, the relative configuration of compound **1** was ascertained. Subsequently, a comparative analysis between eight potential configurations and experimental ECD data was conducted through calculations. Ultimately, it was deduced that the absolute configuration of compound **1** is 4*S*, 5*R*, 6*R*, 7*S*, and 12*S*. Thus, the structure of **1** was determined, and it was denoted as asperpentenone B (**1**).

Compound **2**, a brown solid, was determined to have the molecular formula C_16_H_16_O_6_ based on the (+) HRESIMS at *m*/*z* 305.1027 [M + H]^+^, and the analysis of ^13^C NMR revealed nine degrees of unsaturation (Figures S10 and S13). A comprehensive examination of its ^1^H NMR data (Figure S9) in DMSO-*d*_6_ revealed the presence of three methyls (*δ*_H_ 1.38 (d, *J* = 6.7 Hz, 7-CH_3_); 2.20 (s, 10-CH_3_); and 2.07 (s, 12-CH_3_)); one oxymethylene group (*δ*_H_ 4.35 (s, H-1)); and one alkene proton (*δ*_H_ 6.33 (s, H-3)). The diagnostic NMR data suggested the presence of six benzene carbons (*δ*_C_ 145.4, 103.6, 152.9, 108.1, 150.3, and 104.0) and a carbonyl group (*δ*_C_ 170.6). A comparison of its NMR spectroscopic data with compound **5** showed many similarities. The planar structure of **2** was deduced via 2D NMR spectra (Figures S11 and S12). In the HMBC spectrum (Figure 2), two tertiary methyl groups (*δ*_H_ 2.07, H-16, and 2.20, H-15, each 3H, s) correlating with C-13, C-12, and C-11 as well as C-11, C-10, and C-9, respectively. The multiple HBMC correlations of H-14/C-7, C-8, C-6, H-7/C-14, C-8, C-6, C-5, and C-13 indicated the position of C-7 and C-14 (Figure 2). The absolute configuration of compound **2** was further confirmed through ECD calculations (Figure 4). The compound was analyzed in two different configurations, as depicted in Figure 3. Comparing the calculated ECD with experimental ECD (Figure S14), it can be concluded that compound **2**, possesses an absolute configuration of 7*R*. Consequently, the elucidated structure of compound **2** was unequivocally identified as asperpentenone C (**2**).

Compound **3**, a brown oil, was determined to have a molecular formula of C_15_H_16_O_5_ based on the (+) HRESIMS ion at *m*/*z* 277.1071 [M + H]^+^, 299.0897 [M + Na]^+^ (Figure S19), indicating the presence of eight degrees of unsaturation. The detailed analysis of the ^1^H NMR data (Figure S15) of **3** revealed the presence of one methyl (*δ*_H_ 1.75 (dd, *J* = 6.7, 1.6 Hz, H-15)); four alkene protons (*δ*_H_ 5.71 (m, H-11); 6.31 (dd, *J* = 15.2, 10.4 Hz, H-12); 6.09 (ddd, *J* = 15.0, 10.3, 1.9 Hz, H-13); and 5.77 (m, H-14)); two methylene groups (*δ*_H_ 3.46 (s, H-2); 3.26 (dd, *J* = 15.4, 8.9 Hz, H-9); and 2.87 (dd, *J* = 15.3, 8.2 Hz, H-9)), as well as one oxyhypomethyl (*δ*_H_ 5.15 (q, *J* = 8.1 Hz, H-10)). The presence of fifteen carbon signals of **3**, including a carbonyl group (*δ*_C_ 176.8) and six benzene carbons (*δ*_C_ 129.4, 110.8, 150.9, 104.0, 140.9, and 140.9), was deduced with the obtained NMR spectra (^13^C NMR and HSQC) (Figures S16 and S17). In comparison with the literature, its structure exhibited a close relationship with the known compound asperfuran [11], differing only in the presence of the ethylic acid at C-3, which aligned with the molecular formula. This assertion was further supported by the key HMBC correlations from H-2 to C-3, C-8 C-4, and C-1 (Figure 2 and Figure S18). The absolute configuration of **3** was additionally confirmed through ECD calculations (Figure 4). The ECD calculation of compound **3** was performed for two different conformations, and by comparing the experimental ECD curves (Figure S20), it was determined that the absolute configuration is 10*S*. Therefore, the depicted structure of **3** is firmly established.

**Figure 5:**
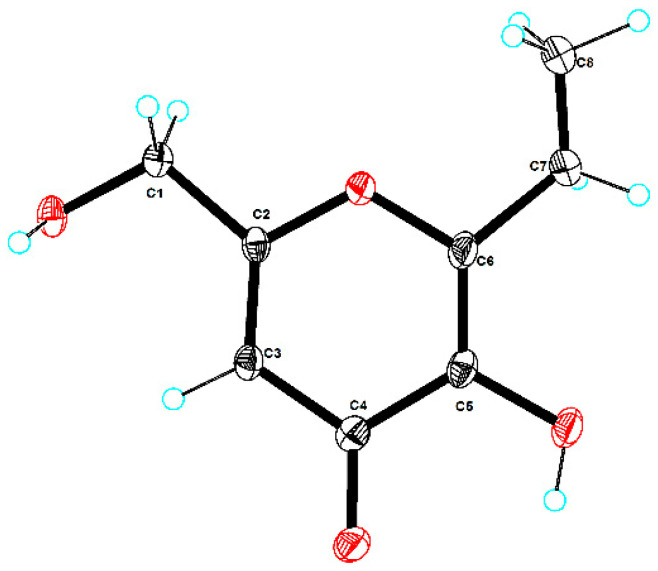
ORTEP drawing of compound **4**.

Compound **4**, red oil, exhibited a molecular formula C_8_H_10_O_4_ as determined with the (+) HRESIMS ion at *m*/*z* 171.0654 [M + H]^+^ (Figure S25), indicating four degrees of unsaturation. A comprehensive analysis of ^1^H NMR data of **4** revealed the presence of one methyl group (*δ*_H_ 1.16, (t, *J* = 7.5 Hz, H-8)) and two methylene groups (*δ*_H_ 4.31, (s, H-1) and 2.14, (q, *J* = 7.5 Hz, H-7)). The ^13^C NMR data of **4** showed the presence of eight carbons, including a carbonyl group (*δ*_C_ 173.5) and one oxymethylene group (*δ*_C_, 59.5). The diagnostic NMR data (Table 2, Figures S21–S24) exhibited several similarities to compound **8** [12]. The only discernible difference was the identification of an ethyl group located at C-6, which was confirmed by the corresponding NMR signals (*δ*_H_ 2.14 and 1.16; *δ*_C_ 21.0 and 11.0), as well as key HMBC correlations from H-7 to C-6 and C-8 (Figure 2). Ultimately, the structure of **4** was further validated through single-crystal X-ray diffraction analysis, with a perspective ORTEP plot depicted in Figure 5. Thus, based on these findings, the structure of compound **4** was unequivocally determined as asperpentenone E.

The other twelve compounds (Figure 1) were elucidated as kojicone A (**5**) [13], 6,8-dihydroxy-3-((1*E*,3*E*)-penta-1,3-dien-1-yl)isochroman-1-one (**6**) [14], germicidin C (**7**) [15], 5-hydroxy-2-hydroxymethyl-4H-pyran-4-one (**8**) [12], sydowione B (**9**) [16], paecilpyrone A (**10**) [16], phomaligol A (**11**) [17], vanillic acid (**12**) [18], methyl-hydroxyphenylaceta (**13**) [19], trans-ferulic acid (**14**) [20], nicotinic acid (**15**) [21], and 7,9-dihydroxy-3-(1H-indol-3-ylmethyl)-8-methoxy-2,3,11,11a-tetrahydro-6H-pyrazino[1,2-b]isoquinoline-1,4-dione (**16**) [22] (Supplementary Materials).

All of these compounds were subjected to screening for their inhibitory effects on acetylcholinesterase (AChE), pancreatic lipase (PL), and neuraminidase (NA) in vitro. Initially, we carried out comprehensive screening for the enzyme inhibitory activities of AChE, PL, and NA at a concentration of 50 μg/mL. This initial screening was crucial as it helped us to identify the promising compounds for further research. The compounds chosen for IC_50_ value determination were those exhibiting an inhibition rate exceeding 50%. The outcomes of the bioassay results revealed the activity of compounds **1**, **10**, **13**, and **14** against AChE, with inhibition rates ranging from 20% to 26% at a concentration of 50 μg/mL. This result suggested that these compounds could potentially slow down the breakdown of acetylcholine in the brain, which is a neurotransmitter that plays a crucial role in memory and cognitive function. We identified three compounds with initial screening inhibition rates exceeding 50% and therefore determined the IC_50_ value for each of them. The standard curve of these compounds was established by measuring the inhibition rate of different concentrations, and the equation of the standard curve was derived. Subsequently, the concentration of the compound with an inhibition rate of 50%, known as the IC_50_ value, was determined. Ultimately, compound **3** demonstrated weak activity against PL, with an IC_50_ value of 127.11 μM, compared with the positive control, which was 0.078 μΜ. Orlistat and tacrine were used as positive controls in the PL and AChE inhibitory assays, respectively. Compounds **2** and **14** showed anti-neuraminidase properties, with IC_50_ values of 31.28 μM and 73.63 μM, respectively, compared with the positive control, with an IC_50_ value of 20 μM (oseltamivir phosphate) (Table 3). The purpose of this evaluation was to identify potential candidates for further research and development, with the ultimate goal of harnessing their potential inhibitory properties to treat various medical conditions.

**Figure 6:**
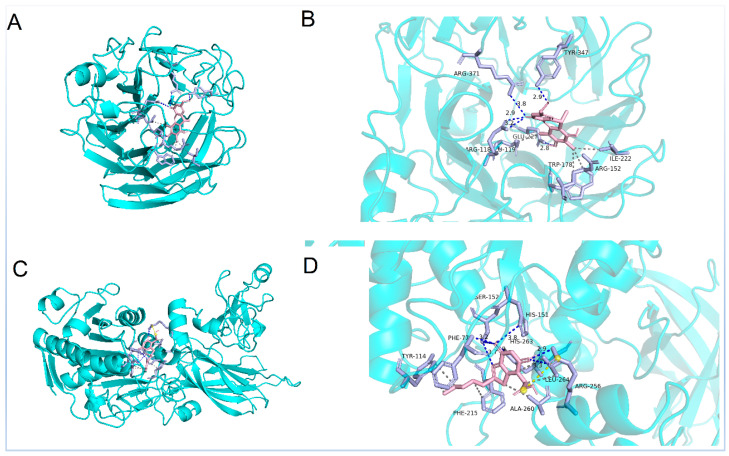
Molecular docking predicted the binding interactions of compounds **2** (**A**,**B**) and **3** (**C**,**D**). Blue dotted line: hydrogen bond; grey dotted line: hydrophobic interaction; yellow dotted line: salt bridge.

Molecular docking analysis was conducted to gain insights into the molecular interactions between **3** and PL, and between **2** and NA, in order to further comprehend the interaction between these compounds and proteins. This comprehensive study aimed to enhance our understanding of the intricate relationship between these compounds and proteins, shedding light on their binding mechanisms, and ultimately aiding in the development of new pharmaceutical agents. Based on the results, compound **3** was found to be favorably accommodated within the binding cleft with similar anchoring conformations, exhibiting a binding free energy (designed as S value) of 8.5 kcal/mol. Compound **3** demonstrated an interaction with PL protein (PDB code: 1PLB), primarily forming hydrogen bonds with amino acid and residues PHE77, HIS151, ARG256, and SER152 within the target protein at distances ranging from 2.9 Å to 3.8 Å. Additionally, hydrophobic interactions were observed with TYR114, PHE215, ALA260, and LEU264, while a salt bridge interaction occurred with ARG256 (Figure 6). On the other hand, compound **2** displayed binding free energy of 7.9 kcal/mol and interacted with NA protein (PDB code: 2HU4), mainly through hydrogen bonds with amino acid and residues ARG118, GLU227, TYR347, and ARG371 within the target protein at distances ranging from 2.8 Å to 3.8 Å, hydrophobic interactions were observed with GLU119, ARG152, TRP178, and ILE222 (Figure 6). These findings offer a rational explanation for the intricate interactions between compounds and enzymes, which play a crucial role in numerous biochemical processes within living organisms.

## 3. Materials and Methods

### 3.1. Standardized Experimental Procedures

NMR spectra were acquired utilizing a state-of-the-art instrument, either a Bruker AC 500 or an AVANCE III HD 700 NMR spectrometer (Bruker, Fällanden, Switzerland), and TMS served as an internal standard. HR-ESI-MS data were obtained by employing the cutting-edge Bruker microTOF-QII mass spectrometer in positive/negative ion mode (Bruker, Fällanden, Switzerland). Semi-preparative HPLC was conducted on a YMC-pack ODS-A column measuring 10 × 250 mm and consisting of particles that were 5 µm in size (Kyoto, Japan). Column chromatography was performed on silica gel with particle sizes ranging from 100 to 200 mesh grade and 200 to 300 mesh grade, from Jiangyou Silica Gel Development Co., Yantai, China. TLC was conducted using silica gel GF254 plates (0.4−0.5 mm) from Qingdao Marine Chemical Factory in Qingdao, China. Spots were visualized under UV light. Acetylcholine esterase (Solarbio, Beijing, China), Thiodide acetylcholine, DTNB, and Tacrine (Sigma, St. Louis, MO, USA) were used for evaluating anti-acetylcholinesterase activity. A neuraminidase inhibitor screening kit was utilized for assessing anti-neuraminidase activity.

### 3.2. Fungal Material

The soft coral BH2, obtained from Beihai, Guangxi Province, China, in November 2018, yielded the fungal strain *Aspergillus austwickii* SCSIO41227. Through morphological examination and sequence analysis of the internal spacer (ITS) regions of rDNA, it was determined that this strain belongs to *Aspergillus austwickii*. A voucher specimen has been deposited in the CAS Key Laboratory of Tropical Marine Bioresources and Ecology, South China Sea Institute of Oceanology, under the Chinese Academy of Sciences in Guangzhou, China.

### 3.3. Fermentation, Extraction, and Isolation Processes

The strain was cultivated on MB agar plates at a temperature of 25 °C for a duration of seven days. A seed medium consisting of malt extract (15 g), sea salt (10 g), and distilled water (1000 mL) was inoculated with *Aspergillus austwickii* SCSIO41227 and incubated on a rotating shaker (180 rpm/s) at the same temperature for 72 h. The strain *Aspergillus austwickii* SCSIO41227 was cultured in rice medium flasks (67 in total) containing rice (200 g/flask), sea salt (7.0 g/flask), and distilled water (200 mL/flask). These flasks were statically incubated under a normal 24 h cycle at the aforementioned temperature. After a period of 35 days, the rice medium was soaked in EtOAc solution (600 mL/flask), fragmented into small pieces, and subjected to sonication for a duration of 20 min. Subsequently, the mixture was transferred to fermentation vats, which underwent four rounds of extraction using EtOAc solvent, followed by concentration under reduced pressure, yielding a crude extract weighing 144 g.

The crude extract was combined with silica gel of 100–200 mesh size, and a gradient separation technique was employed using medium-pressure column chromatography with the 200−300 mesh silica gel. Stepwise elution with CH_2_Cl_2_–MeOH (*v*/*v* 100:1, 80:1, 50:1, 20:1, 0:1, 500 mL each) was performed on a silica gel column to yield ten subfractions (Frs.1−10), as determined via TLC analysis. Among these fractions, Fr.2 underwent reversed-phase intermediate pressure chromatography (ODS) with MeOH–H_2_O (*v*/*v*, 1:9-10:0, 500 mL each), resulting in the isolation of ten additional subfractions (Fr.2.1−2.10). Fr.2.4 was obtained through semi-prepared liquid-phase chromatography (HPLC) (MeOH/H_2_O = 53/47, adding 0.04% FA, 3 mL/min, *t*_R_ = 18 min) to obtain **11** (8.80 mg). Fr.2.10 was purified via HPLC (MeOH/H_2_O = 65/35, adding 0.06% FA, 3 mL/min, *t*_R_ = 26 min) to obtain **1** (22.29 mg) and **6** (*t*_R_ = 18 min, 2.89 mg). Fr.3 was fractionated via ODS using MeOH–H_2_O (*v*/*v*, 1:9–10:0, 500 mL each) to acquire ten subfractions (Fr.3.1−3.10). Fr.3.1 was further purified via HPLC (MeOH/H_2_O = 45/55, adding 0.04% FA, 2.5 mL/min, *t*_R_ = 19 min) to obtain **13** (3.08 mg), and **12** was obtained using another HPLC method (MeOH/H_2_O = 25/75, adding 0.04% FA, 3 mL/min, *t*_R_ = 24 min, 39.2 mg). The fraction of Fr.3.2 using HPLC (MeCN/H_2_O = 34/66, adding 0.06% FA, 3 mL/min, *t*_R_ = 8 min) yielded **14** (12.36 mg). Fr.3.3 was prepared via HPLC (MeOH/H_2_O = 40/60, adding 0.06% FA, 3 mL/min, *t*_R_ = 30 min), resulting in the isolation of **7** (13.9 mg) and **10** (*t*_R_ = 32 min, 4.29 mg). Fr.3.10 was purified using HPLC (MeOH/H_2_O = 70/30, adding 0.06% FA, 3 mL/min, *t*_R_ = 10 min) to obtain **5** (21.95 mg). Fr.4 underwent ODS using a mixture of MeOH-H_2_O (*v*/*v*, 1:9–10:0, 500 mL each), resulting in ten subfractions (Fr.4.1−4.10). Fr.4.1 was obtained by performing HPLC (MeCN/H_2_O = 10/90, adding 0.04% FA, 3 mL/min, *t*_R_ = 14 min) to yield **4** (10.08 mg) and **15** (*t*_R_ = 8 min, 2.25 mg). Fr.4.2 was purified via HPLC (MeOH/H_2_O = 55/45, adding 0.06% FA, 3 mL/min, *t*_R_ = 21 min) to obtain **9** (5.98 mg). Fr.5 underwent ODS using varying ratios of MeOH-H_2_O (*v*/*v*, 1:9−10:0, 500 mL each) to obtain ten subfractions (Fr.5.1−5.10). Fr.5.7 was further purified via HPLC (MeOH/H_2_O = 60/40, adding 0.06% FA, 3 mL/min, *t*_R_ = 21 min) to obtain **3** (3.22 mg). Fr.7 was divided into eleven subfractions (Fr.7.1–7.11) through ODS with MeOH/H_2_O (*v*/*v*, 1:9−10:0, 500 mL each). Fr.7.10 was further purified via HPLC (MeOH/H_2_O = 41/59, 3 mL/min, *t*_R_ = 10 min) to obtain **16** (3.22 mg). Fr.7.11 was prepared via HPLC (MeOH/H_2_O = 45/55, adding 0.04% FA, 3 mL/min, *t*_R_ = 20 min) to yield compound **2** (2.93 mg).

*Asperpentenone B* (**1**): brown oil; [α${]}_{D}^{25}$ + 23.4 (c 0.05, CH_3_OH); UV λ_max_ (Δ*ε*) 262 (1.09) nm; ECD (0.3 mg/mL, MeOH) λ_max_ (Δ*ε*) 220 (+4.29), 258 (+0.04), 265 (+0.55) 285 (−0.76) nm; ^1^H NMR (DMSO-*d*_6_, 500 MHz), ^13^C NMR (DMSO-*d*_6_, 125 MHz) data (see Table 1); HRESIMS *m/z* 285.1698 [M + H]^+^ (calcd. for C_15_H_25_O_5_, 285.1697), 307.1513 [M + Na]^+^ (calcd. for C_15_H_24_NaO_5_, 307.1516).

*Asperpentenone C* (**2**): brown solid; [α${]}_{D}^{25}$ − 3.4 (c 0.05, CH_3_OH); UV λ_max_ (Δ*ε*) 200 (1.68), 287 (0.65) nm; ECD (0.3 mg/mL, MeOH) λ_max_ (Δ*ε*) 201 (+2.68), 211 (−0.57) nm; ^1^H NMR (DMSO-*d*_6_, 500 MHz), ^13^C NMR (DMSO-*d*_6_, 125 MHz) data (see Table 1); HRESIMS *m/z* 305.1027 [M + H]^+^ (calcd. for C_16_H_17_O_6_, 305.1020), 327.0838 [M + Na]^+^ (calcd. for C_16_H_16_NaO_6_, 327.0839).

*Asperpentenone D* (**3**): brown oil; [α${]}_{D}^{25}$ − 29.7 (c 0.05, CH_3_OH); UV λ_max_ (Δ*ε*) 210 (1.81), 228 (2.11), 295 (0.41) nm ECD (0.3 mg/mL, MeOH) λ_max_ (Δ*ε*) 210 (+1.64), 222 (−1.22), 231 (−0.08), 241 (−0.83) nm; ^1^H NMR (CD_3_OD, 700 MHz), ^13^C NMR (CD_3_OD, 175 MHz) data (see Table 2); HRESIMS *m/z* 277.1071 [M + H]^+^ (calcd. for C_15_H_17_O_5_, 277.1071), 299.0897 [M + Na]^+^ (calcd. for C_15_H_16_NaO_5_, 299.0890).

*Asperpentenone E* (**4**): red oil; UV λ_max_ (Δ*ε*) 201 (0.79), 217 (1.09), 276 (0.64) nm. ^1^H NMR (DMSO-*d*_6_, 500 MHz), ^13^C NMR (DMSO-*d*_6_, 125 MHz) data (see Table 2); HRESIMS *m/z* 171.0654 [M + H]^+^ (calcd. for C_8_H_11_O_4_, 171.0652), 193.0472 [M + Na]^+^ (calcd. for C_8_H_10_NaO_4_, 193.0471).

Crystallographic data for compound **4**: Moiety formula was C_8_H_10_O_4_ (MW = 170.16), and the crystal size was 0.19 × 0.11 × 0.1 mm^3^, exhibiting a tetragonal structure with space group I4_1_/a, and unit cell dimensions were a = 21.5062 (6) Å, b = 21.5062 (6) Å, and c = 7.0693 (3) Å, resulting in a volume of V = 3269.7 (2) A^3^. This accommodated Z = 16 molecules per unit cell at a calculated density of *ρ*_calc_ = 1.383 g/cm^3^ under temperature T = 100.0 (2) K, using Cu K*α* radiation with μ = 0.950 mm^−1^. The absorption coefficient was determined by measuring a total of 3669 reflections, 1615 of which were independent reflections, yielding *R*_int_ = 0.0329 and *R*_sigma_ = 0.0375. Final *R* indexes (I ≥ 2σ (I)) were found to be *R*_1_ = 0.0548 and *wR*_2_ = 0.1495, and when considering all data, the final *R* indexes were *R*_1_ = 0.0709 and *wR*_2_ = 0.1567. The largest diff peak and hole were observed as 0.32 and −0.28 e Å^−3^.

### 3.4. X-ray Crystallographic Analysis

Compound **4** was obtained as colorless crystals through the process of slow evaporation at room temperature in a mixture of MeOH and CH_2_Cl_2_ (9:1). The crystal’s information was collected using Cu Kα radiation on an XtalLAB PRO single-crystal diffractometer. The X-ray crystal structure of **4** was determined using SHELXS97, expanded by difference Fourier techniques, and refined through full-matrix least-square calculation. Crystallographic data of **4** have been deposited at the Cambridge Crystallographic Data Centre (deposition number: 2301520). These data can be acquired free of charge by containing CCDC at 12 Union Road, Cambridge CB21EZ, UK.

### 3.5. ECD Calculation

The structures of compounds **1**–**3** underwent extensive conformational searches utilizing the advanced Spanran’14 software with the MMFF method [23]. The conformers that had a Boltzmann distribution exceeding 5% were selected for ECD calculations, which were performed using the Gaussian 09 software (Gaussian inc., Wallingford, CT, USA). Stable conformers were initially optimized in MeOH at the B3LYP/6-31G level before performing an overall theoretical calculation of ECD in MeOH using time-dependent density functional theory at the B3LYP/6-311G (d, p) level. Finally, ECD spectra were ultimately generated using Gaussian view 6.0 (Gaussian inc., Wallingford, CT, USA) with a half-bandwidth of 0.33 eV and Origin 2021 software (Origin Lab, Northampton, MA, USA). This was accomplished by considering the Boltzmann-calculated contribution of each conformer after UV correction.

### 3.6. Bioassays

The inhibitory effects of **1**–**16** on AChE were assessed in vitro at a concentration of 50 μg/mL according to the modified Ellman method [24]. To prepare the AChE solution, it was dissolved in phosphate buffer (pH 8.0), achieving a concentration of 0.1 U/mL. These compounds and enzyme buffer were combined in 96-cell plates and incubated for 20 min at a temperature of 30 °C. Then, we introduced 5,5’-dithibis (2-nitrobenzoic acid), and acetylthiocholine iodide for 30 min at 30 °C. The inhibition rate of AChE was determined by measuring the degradation of acetylthiocholine iodide to thiocholine and acetic acid at 405 nm using a microplate reader. Similarly, PL activity was also evaluated in vitro using our previously established methods, with measurements conducted in 96-cell plates. The experimental procedures and data analysis followed our previously described protocol. For testing the inhibitory effects of NA, we employed a neuraminidase inhibitor screening kit as per its manufacturer’s instructions.

### 3.7. Molecular Docking

The molecular docking simulation was conducted using AutoDock Tools (ADT 1.5.6) software [25] (Scripps, San Diego, CA, USA). The initial models for PL (PDB code:1LPB) [26] and NA (PDB code: 2HU4) [27] obtained from the Protein Data Bank were utilized after removing all water molecules and organic small molecules, while the ligand structures were generated in ChemBioOffice 20.0 (PerkinElmer Informatics, Waltham, MA, USA), followed by MM2 calculations to minimize the conformation energy. The dimensions of the grid box used were as follows: 78.05 × 78.05 × 78.05; 1.133 × 50.29 × 100.51 with centers at x: −6.863, y: 29.851, z: 38.093 and x: 110.95, y: 109.19, z: 66.92, respectively. The default settings and calculations were applied for other docking parameters, while PyMol software version 2.4.0 (Schrödinger, New York, NY, USA) was employed for analyzing the docking results.

## 4. Conclusions

Two novel polyketides, asperpentenones B (**1**) and C (**2**), along with a newly discovered asperfuran, asperpentenone D (**3**), and a novel kojic acid dipalmitate, asperpentenone E (**4**), were isolated from coral-derived fungus *Aspergillus austwickii* SCSIO41227. Twelve additional compounds were also identified from this fungus. The planar structures and absolute configurations of these compounds were determined through comprehensive spectroscopic analysis using experimental and calculated ECD data, which were then compared with the existing data in the literature. Several of the isolated compounds displayed enzyme inhibitory activity against AChE, PL, and NA. Compounds **2** and **14** showed anti-neuraminidase activity with IC_50_ values of 31.28 μM and 73.64 μM, respectively, compared with the positive control, with an IC_50_ value of 20 μM. Compounds **1**, **10**, **13**, and **14** showed activity against AChE with an inhibition rate ranging from 20% to 26%, and compound **3** exhibited weak inhibitory effects against PL, with an IC_50_ value of 127.11 μM. In this study, two new compounds exhibited enzyme activities, which provided valuable information for further development of PL and NA inhibitors.

## Figures and Tables

**Table 1:** The NMR data of **1** and **2** (500 and 125 MHz, TMS, *δ* in ppm, DMSO-*d*_6_).

Pos	1		Pos	2	
	*δ*_C_, type	*δ*_H_, (*J* in Hz)		*δ*_C_, type	*δ*_H_, (*J* in Hz)
1	200.6, C		1	59.4, CH_2_	4.35, (s)
2	113.0, C		2	167.5, C	
3	181.2, C		3	111.0, CH	6.33, (s)
4	53.5, CH	3.56, (d, *J* = 1.7 Hz)	4	170.6, C	
5	106.2, C		5	137.5, C	
6	74.4, CH	3.99, (q, *J* = 6.4 Hz)	6	152.0, C	
7	91.7, C		7	28.3, CH	4.12, (q, *J* = 6.6 Hz)
8	7.0, CH_3_	1.68, (d, *J* = 1.5 Hz)	8	104.0, C	
9	12.4, CH_3_	0.89, (t, *J* = 7.4 Hz)	9	150.3, C	
10	51.3, CH_3_	3.04, (s)	10	108.1, C	
11	58.9, CH_3_	4.06, (s)	11	152.9, C	
12	43.1, CH	1.66, (m)	12	103.6, C	
13	22.8, CH_2_	1.06, (m), 1.80 (m)	13	145.4, C	
14	13.8, CH_3_	0.96, (d, *J* = 6.7 Hz)	14	21.4, CH_3_	1.38, (d, *J* = 6.7 Hz)
15	13.0, CH_3_	1.10, (d, *J* = 6.5 Hz)	15	9.6, CH_3_	2.20, (s)
			16	8.7, CH_3_	2.07, (s)

**Table 2:** The NMR data of **3** (700 and 175 MHz) and the NMR data of **4** (500 and 125 MHz) (TMS, *δ* in ppm).

Pos	3 ^a^		Pos	4 ^b^	
	*δ*_C_, type	*δ*_H_, (*J* in Hz)		*δ*_C_, type	*δ*_H_, (*J* in Hz)
1	176.8, C		1	59.5, CH_2_	4.31, (s)
2	34.1, CH_2_	3.46, (s)	2	167.0, C	
3	110.8, C		3	108.9, CH	6.29, (s)
4	150.9, C		4	173.5, C	
5	104.0, CH	6.20, (s)	5	141.1, C	
6	140.9, C		6	152.3, C	
7	140.9, C		7	21.0, CH_2_	2.14, (q, *J* = 7.5 Hz)
8	129.4, C		8	11.0, CH_3_	1.16, (t, *J* = 7.5 Hz)
9	37.2, CH_2_	3.26, (dd, *J* = 15.4, 8.9 Hz) 2.87, (dd, *J* = 15.3, 8.2 Hz)			
10	85.0, CH	5.15, (q, *J* = 8.1 Hz)			
11	130.8, CH	5.71, (m)			
12	133.6, CH	6.31, (dd, *J* = 15.2, 10.4 Hz)			
13	132.0, CH	6.09, (ddd, *J* = 15.0, 10.3, 1.9 Hz)			
14	131.6, CH	5.77, (m)			
15	18.2, CH_3_	1.75, (dd, *J* = 6.7, 1.6 Hz)			

^a^ Recorded in CD^3^OD; ^b^ Recorded in DMSO-*d_6_*.

**Table 3:** The inhibitory activities of the compounds against pancreatic lipase and neuraminidase.

Compounds	IC_50_ Value	
	PL	NA
**3**	127.11 μM	-
**2**	-	31.28 μM
**14**	-	73.64 μM

## Data Availability

Not applicable.

## Supplementary Materials

The following supporting information can be downloaded at: https://www.mdpi.com/article/10.3390/md21110567/s1, The ITS sequence data of *Aspergillus austwickii* SCSIO41227: Figures S1–S26: ^1^H, ^13^C-NMR, HSQC, HMBC, ^1^H-^1^H COSY, HRESIMS, and CD spectra of compounds **1**–**4**. The physicochemical data of compounds **5**–**16**.
