# Supplementary material for: Secondary Metabolites from the Coral-Derived Fungus Aspergillus austwickii SCSIO41227 with Pancreatic Lipase and Neuraminidase Inhibitory Activities
Source: Mar Drugs. 2023 Oct 29;21(11):567. doi: 10.3390/md21110567 (PMC10672402; doi:10.3390/md21110567)

SUPPLEMENTARY MATERIAL

Secondary Metabolites from the Coral-Derived Fungus *Aspergillus austwickii* SCSIO41227 with Pancreatic Lipase and Neuraminidase Inhibitory Activities

Ying Chen [1,2], Yan Chun He [1,2], Xiaoyan Pang [1], Xuefeng Zhou [1,2], Yonghong Liu [1,2*], and Bin Yang [1,2*]

1 CAS Key Laboratory of Tropical Marine Bio-resources and Ecology/Guangdong Key Laboratory of Marine Materia Medica, South China Sea Institute of Oceanology, Chinese Academy of Sciences, Guangzhou 510301, China; chenying21@mails.ucas.ac.cn (Y.C.); heyanchun22@mails.ucas.ac.cn (Y.H.); xfzhou@scsio.ac.cn (X.Z.)

2 University of Chinese Academy of Sciences, 19 Yuquan Road, Beijing 100049, P. R. China

* Correspondence: yangbin@scsio.ac.cn (B.Yang); yonghongliu@scsio.ac.cn (Y.Liu)

Abstract: The coral-derived fungus *Aspergillus austwickii* SCSIO41227 from Beibu Gulf yielded four previously uncharacterized compounds, namely asperpentenones B–E (**1**–**4**), along with twelve known compounds (**5**–**16**). Their structures were elucidated by HRESIMS and NMR (^{1}H and ^{13}C NMR, HSQC, HMBC), among which the stereo-structure of compounds **1**–**3** was determined by calculated ECD. Furthermore, compounds **1**–**16** were evaluated for their enzyme (acetylcholinesterase (AChE), pancreatic lipase (PL) and neuraminidase (NA)) inhibitory activities. These bioassay results demonstrated that compound **3** exhibited PL inhibitory effect with an IC_{50} value of 127.11 μM. In addition, compounds **2** and **14** displayed noticeable NA inhibitory effects with IC_{50} values of 31.28 and 73.64 μM, respectively. Furthermore, these compounds showed the potential of inhibiting enzymes in silico docking analysis to demonstrate the interactions between compounds and proteins.

Keywords: *Aspergillus austwickii* SCSIO41227; secondary metabolites; pancreatic lipase; neuraminidase; molecular docking

Contents

The ITS gene sequence data of *Aspergillus austwickii* SCSIO41227.
Figure S1. The ^{1}H NMR Spectrum of compound 1 in DMSO-*d*$_6$.
Figure S2. The ^{13}C NMR Spectrum of compound 1 in DMSO-*d*$_6$.
Figure S3. The HSQC Spectrum of compound 1 in DMSO-*d*$_6$.
Figure S4. The HMBC Spectrum of compound 1 in DMSO-*d*$_6$.
Figure S5. The ^{1}H-^{1}H COSY spectrum of compound 1 in DMSO-*d*$_6$.
Figure S6. The NOESY spectrum of compound 1 in DMSO-*d*$_6$.
Figure S7. The HRESIMS spectrum of compound 1 in MeOH.
Figure S8. The UV and experimental ECD spectrum of compound 1.
Figure S9. The ^{1}H NMR Spectrum of compound 2 in DMSO-*d*$_6$.
Figure S10. The ^{13}C NMR Spectrum of compound 2 in DMSO-*d*$_6$.
Figure S11. The HSQC Spectrum of compound 2 in DMSO-*d*$_6$.
Figure S12. The HMBC Spectrum of compound 2 in DMSO-*d*$_6$.
Figure S13. The HRESIMS spectrum of compound 2 in MeOH.
Figure S14. The UV and experimental ECD spectrum of compound 2.
Figure S15. The ^{1}H NMR Spectrum of compound 3 in CD_3OD.
Figure S16. The ^{13}C NMR Spectrum of compound 3 in CD_3OD.
Figure S17. The HSQC Spectrum of compound 3 in CD_3OD.
Figure S18. The HMBC Spectrum of compound 3 in CD_3OD.
Figure S19. The HRESIMS spectrum of compound 3 in MeOH.
Figure S20. The UV and experimental ECD spectrum of compound 3.
Figure S21. The ^{1}H NMR Spectrum of compound 4 in DMSO-*d*$_6$.
Figure S22. The ^{13}C NMR Spectrum of compound 4 in DMSO-*d*$_6$.
Figure S23. The HSQC Spectrum of compound 4 in DMSO-*d*$_6$.
Figure S24. The HMBC Spectrum of compound 4 in DMSO-*d*$_6$.
Figure S25. The HRESIMS spectrum of compound 4 in MeOH.
Figure S26. The UV spectrum of compound 4.
The physicochemical data of the known compounds 5~16.

The ITS gene sequence data of *Aspergillus austwickii*

ACTCGGTAATGATCCTTCCGTAGGGGAACCTGCGGAAGGATCATTACCGAGTGTAGG
GTTCCTAGCGAGCCCAACCTCCCACCCGTGTTTACTGTACCTTAGTTGCTTCGGCGGG
CCCGCCATTCATGGCCGCCGGGGGCTCTCAGCCCCGGGCCCGCGCCCGCCGGAGACA
CCACGAACTCTGTCTGATCTAGTGAAGTCTGAGTTGATTGTATCGCAATCAGTTAAAA
CTTTCAACAATGGATCTCTTGGTTCCGGCATCGATGAAGAACGCAGCGAAATGCGAT
AACTAGTGTGAATTGCAGAATTCCGTGAATCATCGAGTCTTTGAACGCACATTGCGCC
CCCTGGTATTCCGGGGGGCATGCCTGTCCGAGCGTCATTGCTGCCCATCAAGCACGGC
TTGTGTGTTGGGTCGTCGTCCCCTCTCCGGGGGGGACGGGCCCCAAAGGCAGCGGCG
GCACCGCGTCCGATCCTCGAGCGTATGGGGCTTTGTCACCCGCTCTGTAGGCCCGGCC
GGCGCTTGCCGAACGCAAATCAATCTTTTCCAGGTTGACCTCGGATCAGGTAGGGAT
ACCCGCTGAACTTAAGCATATCAATAAAGCGGAG

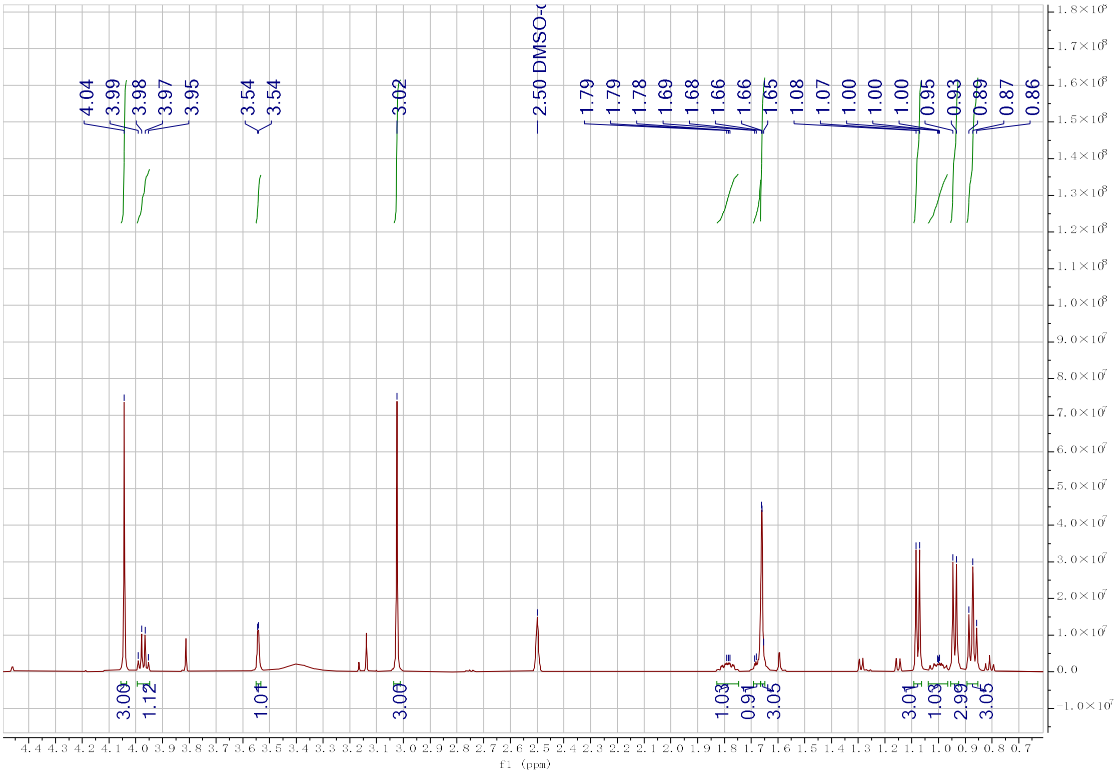

Figure S1. The ^{1}H NMR Spectrum of compound 1 in DMSO-*d*$_6$.

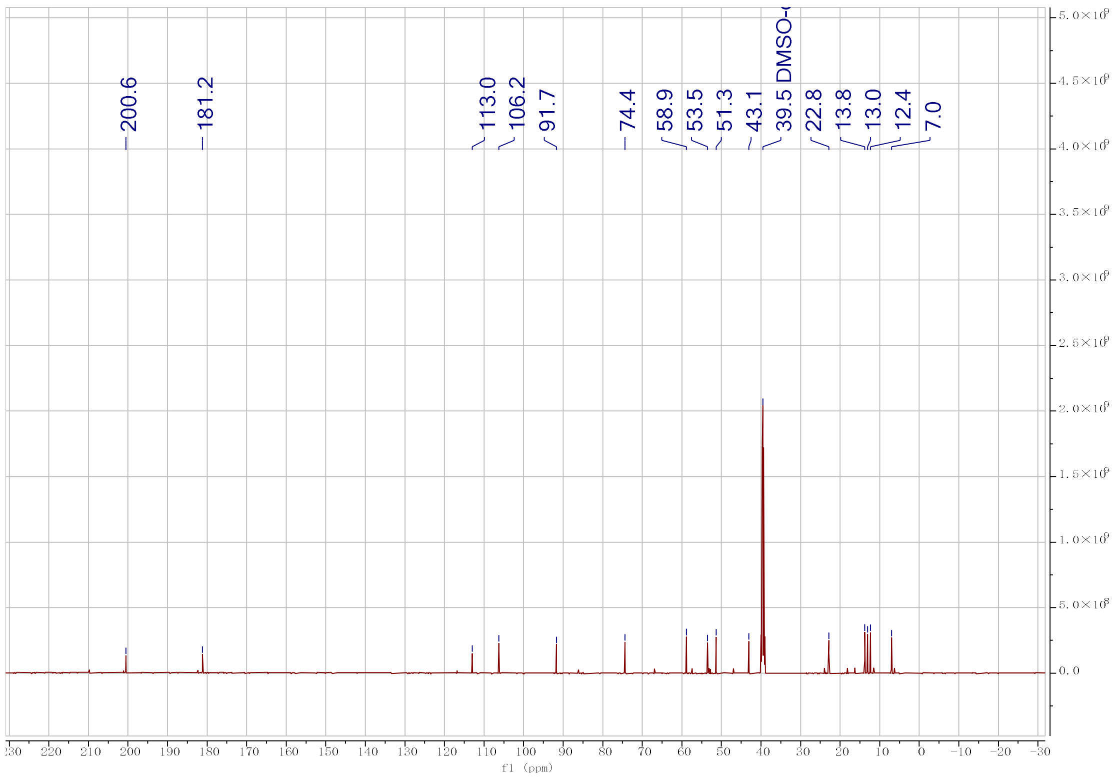

Figure S2. The ^{13}C NMR Spectrum of compound 1 in DMSO-*d*$_6$.

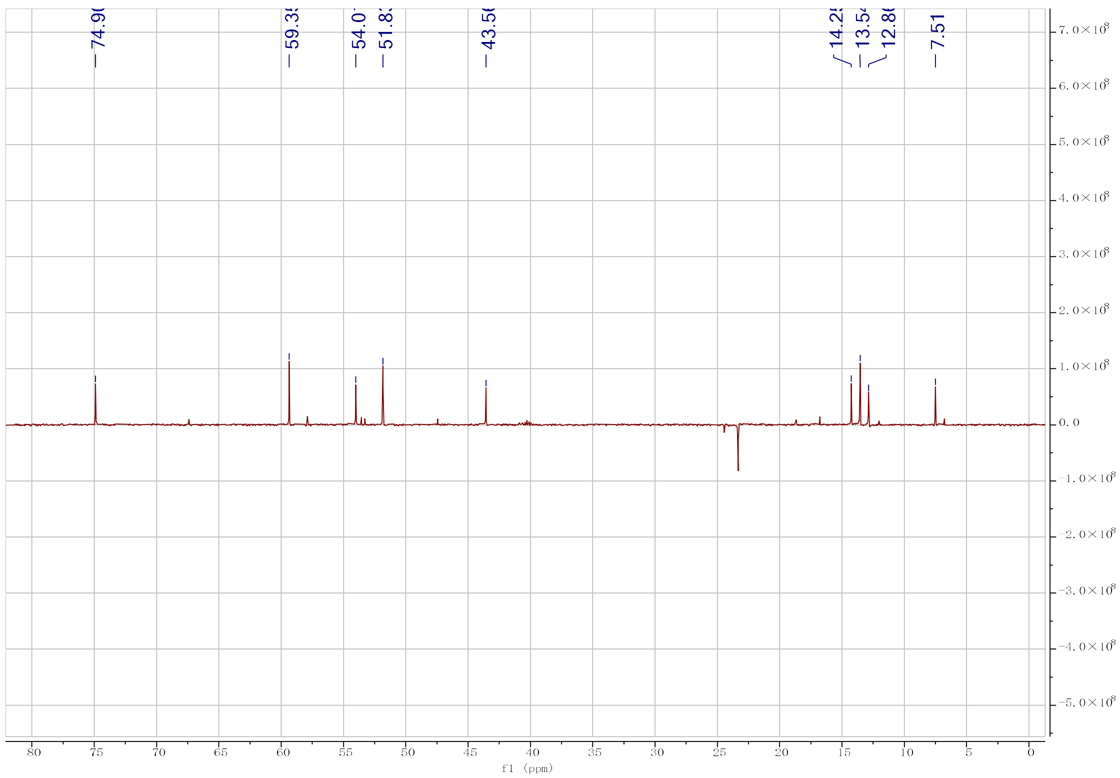

Figure S2. The DEPT Spectrum of compound 1 in DMSO-*d*$_6$.

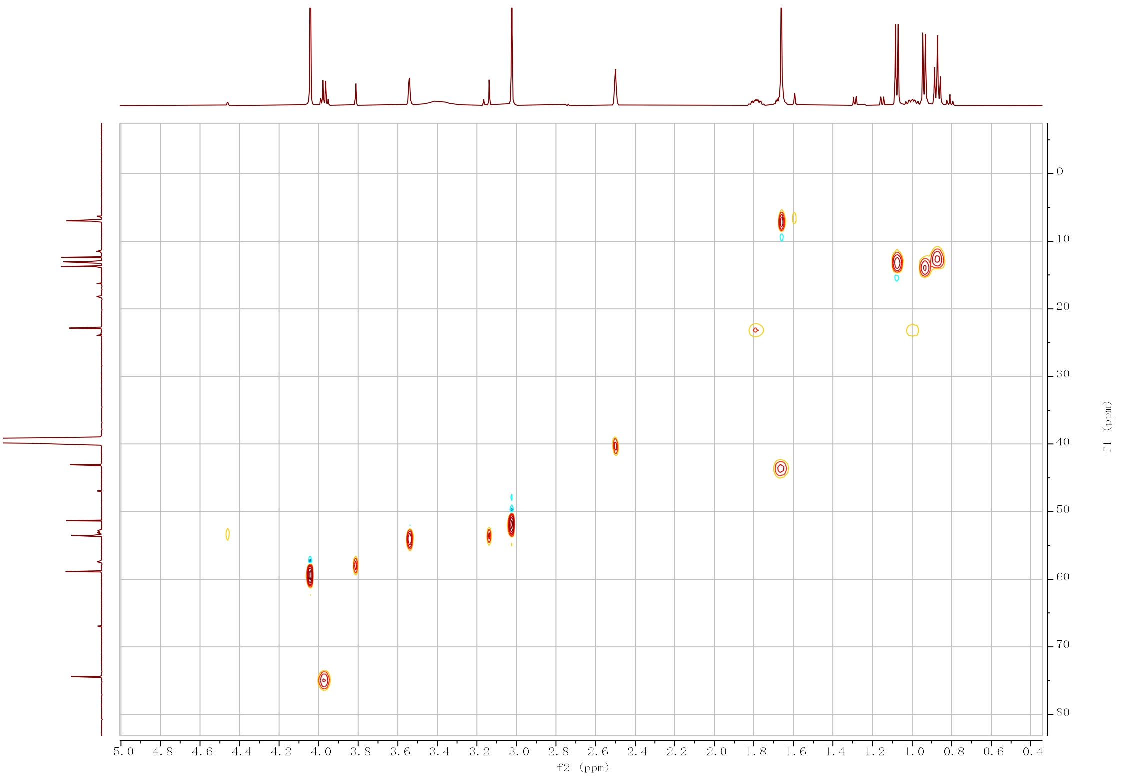

Figure S3. The HSQC Spectrum of compound 1 in DMSO-*d*6.

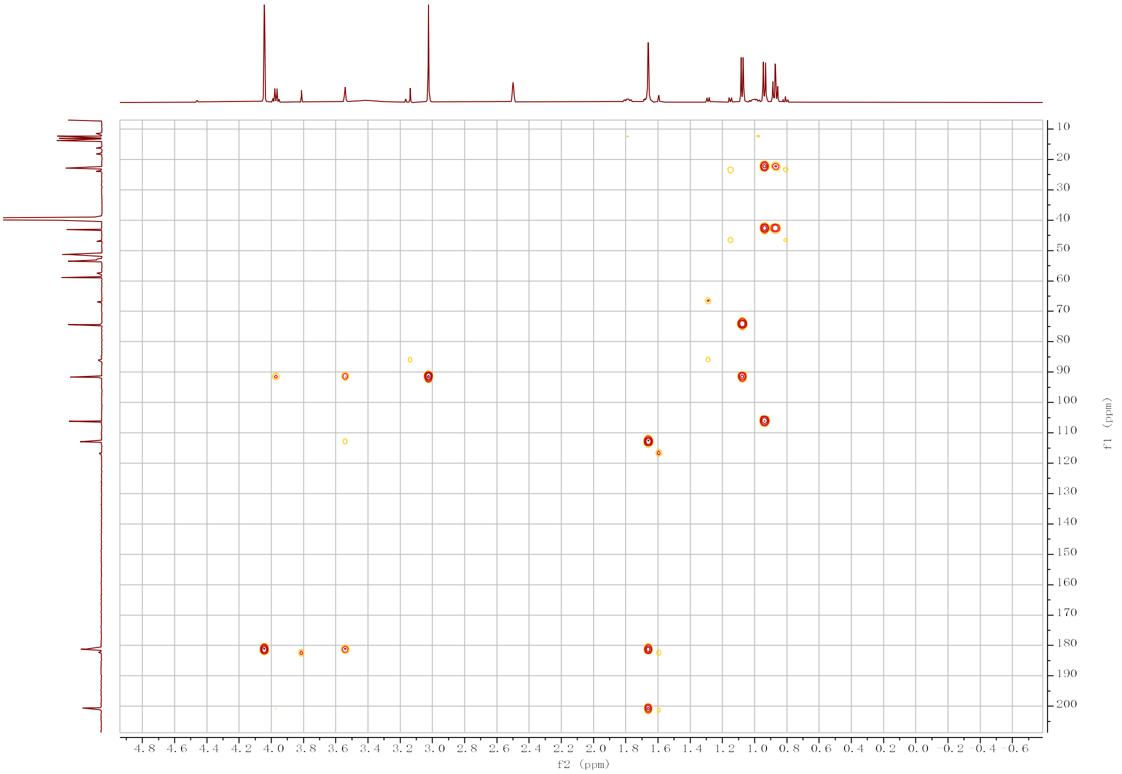

Figure S4. The HMBC Spectrum of compound 1 in DMSO-*d*6.

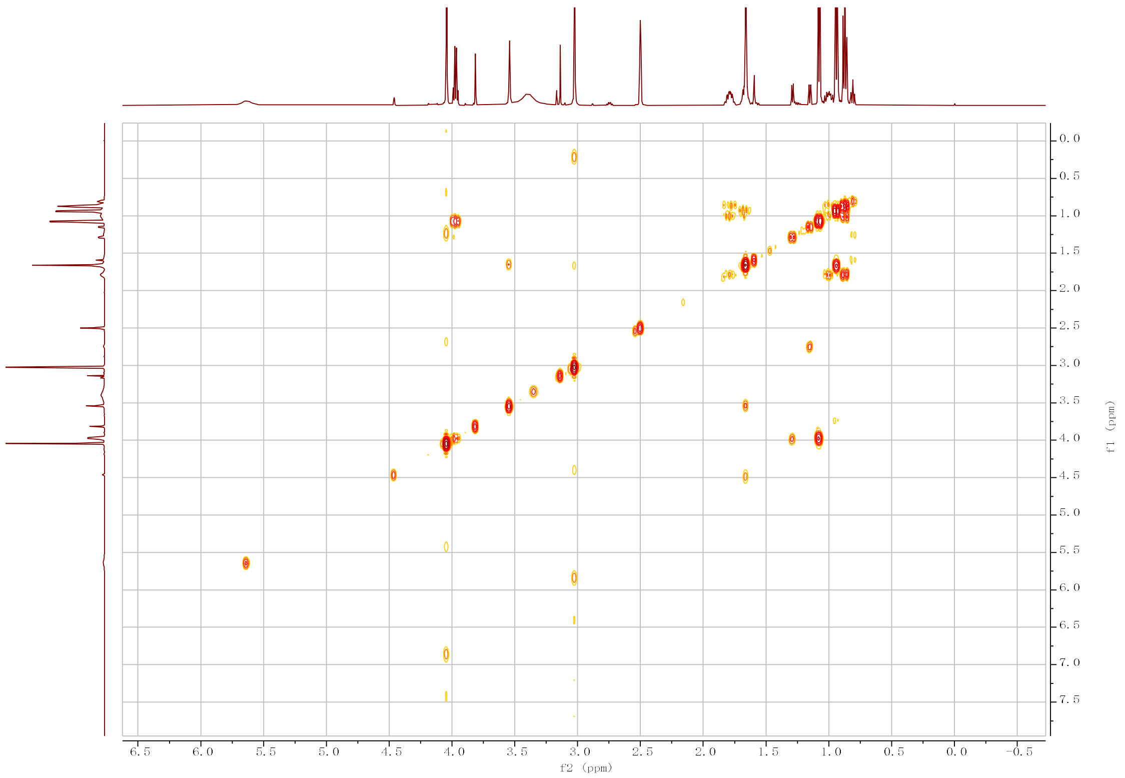

Figure S5. The ^{1}H- ^{1}H COSY spectrum of compound 1 in DMSO-*d*$_6$.

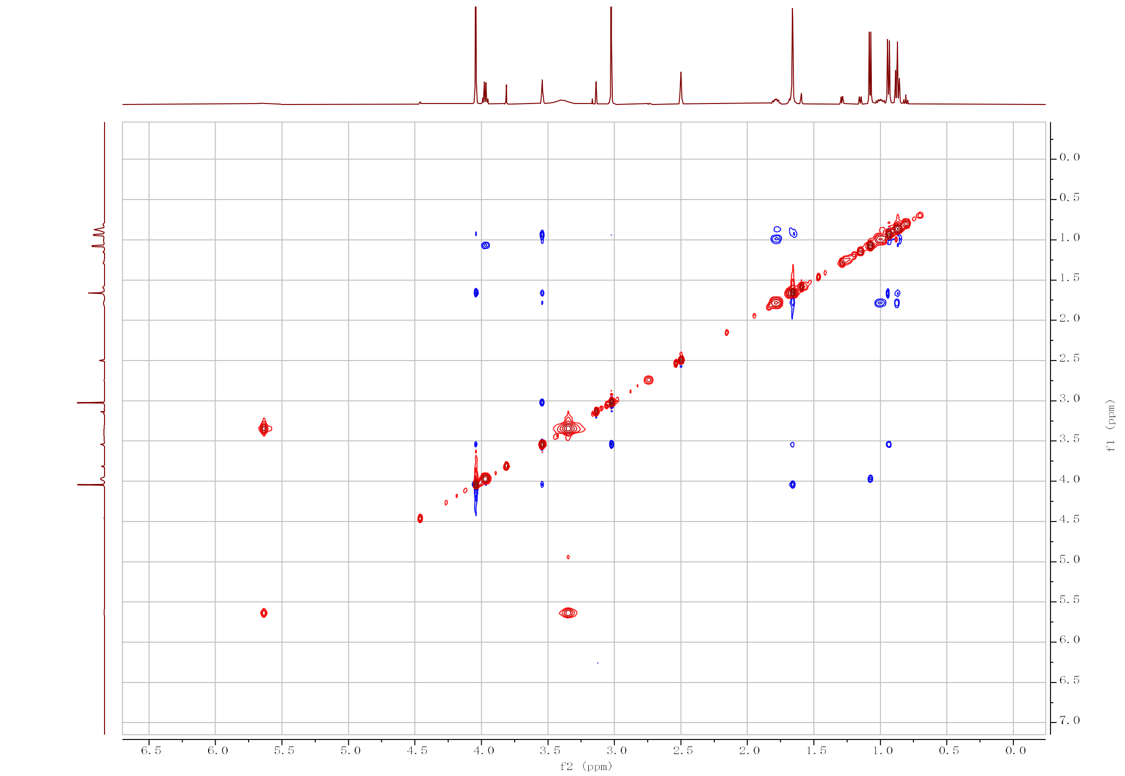

Figure S6. The NOESY spectrum of compound 1 in DMSO-*d*$_6$.

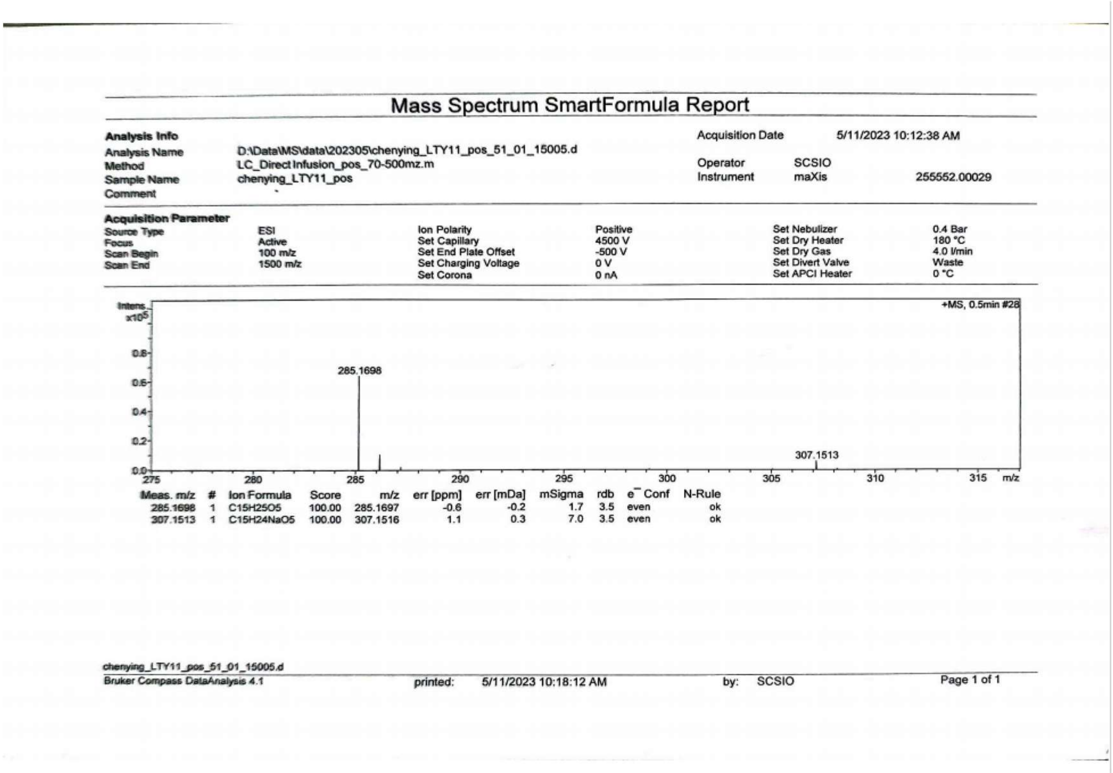

Mass Spectrum SmartFormula Report

Analysis Info

Analysis Name	D:\Data\MS\data\202305\chenying_LTY11_pos_51_01_15005.d	Acquisition Date	5/11/2023 10:12:38 AM
Method	LC_Direct Infusion_pos_70-500mz.m	Operator	SCSIO
Sample Name	chenying_LTY11_pos	Instrument	maXis 255552.00029
Comment			

Acquisition Parameter

Source Type	ESI	Ion Polarity	Positive	Set Nebulizer	0.4 Bar
Focus	Active	Set Capillary	4500 V	Set Dry Heater	180 °C
Scan Begin	100 m/z	Set End Plate Offset	-500 V	Set Dry Gas	4.0 l/min
Scan End	1500 m/z	Set Charging Voltage	0 V	Set Divert Valve	Waste
		Set Corona	0 nA	Set APCI Heater	0 °C

Meas. m/z	#	Ion Formula	Score	m/z	err [ppm]	err [mDa]	mSigma	rdb	e⁻ Conf	N-Rule
285.1698	1	C15H25O5	100.00	285.1697	-0.6	-0.2	1.7	3.5	even	ok
307.1513	1	C15H24NaO5	100.00	307.1516	1.1	0.3	7.0	3.5	even	ok

chenying_LTY11_pos_51_01_15005.d
Bruker Compass DataAnalysis 4.1 printed: 5/11/2023 10:18:12 AM by: SCSIO Page 1 of 1

Figure S7. The HRESIMS spectrum of compound 1 in MeOH.

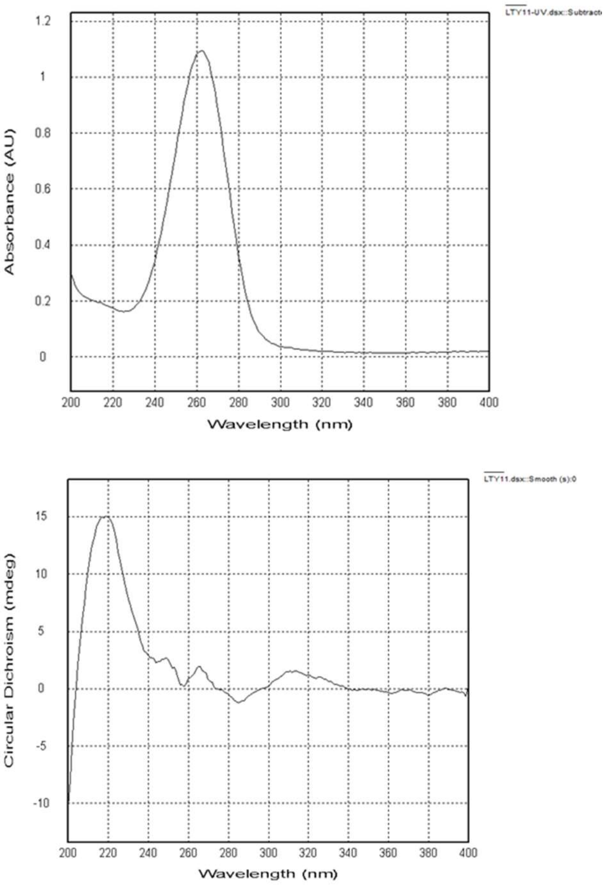

Figure S8. The UV and experimental ECD spectrum of compound 1.

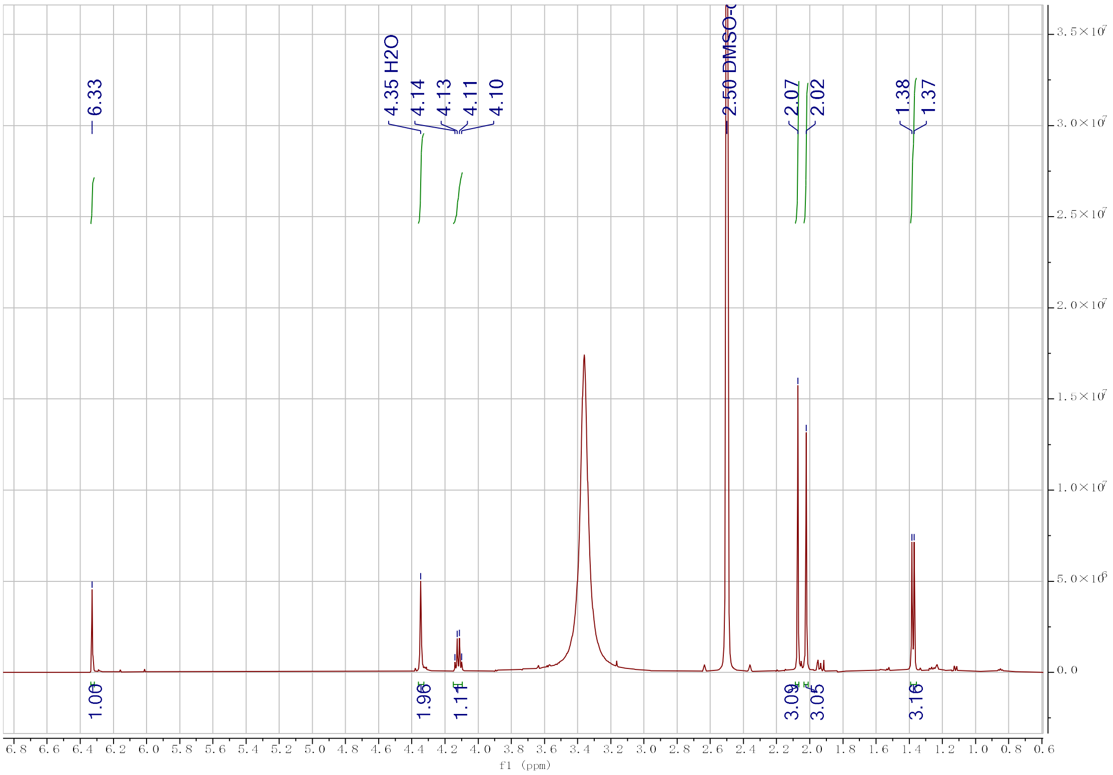

Figure S9. The ¹H NMR Spectrum of compound 2 in DMSO-*d*₆.

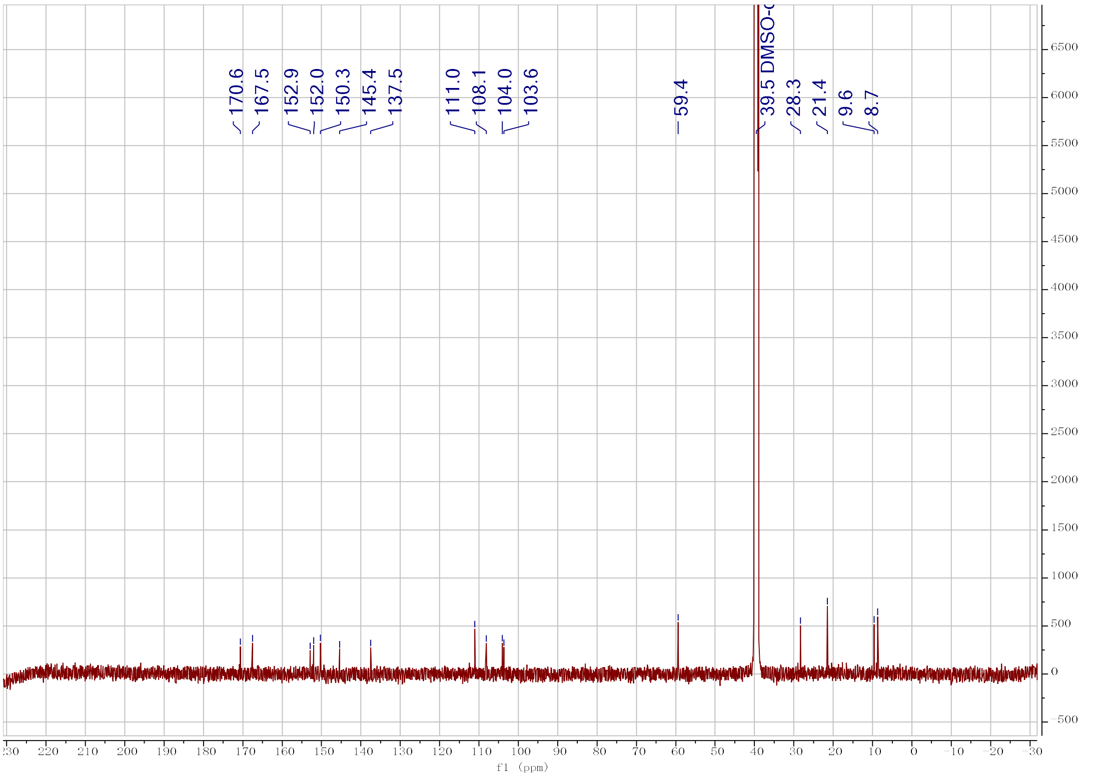

Figure S10. The ¹³C NMR Spectrum of compound 2 in DMSO-*d*₆.

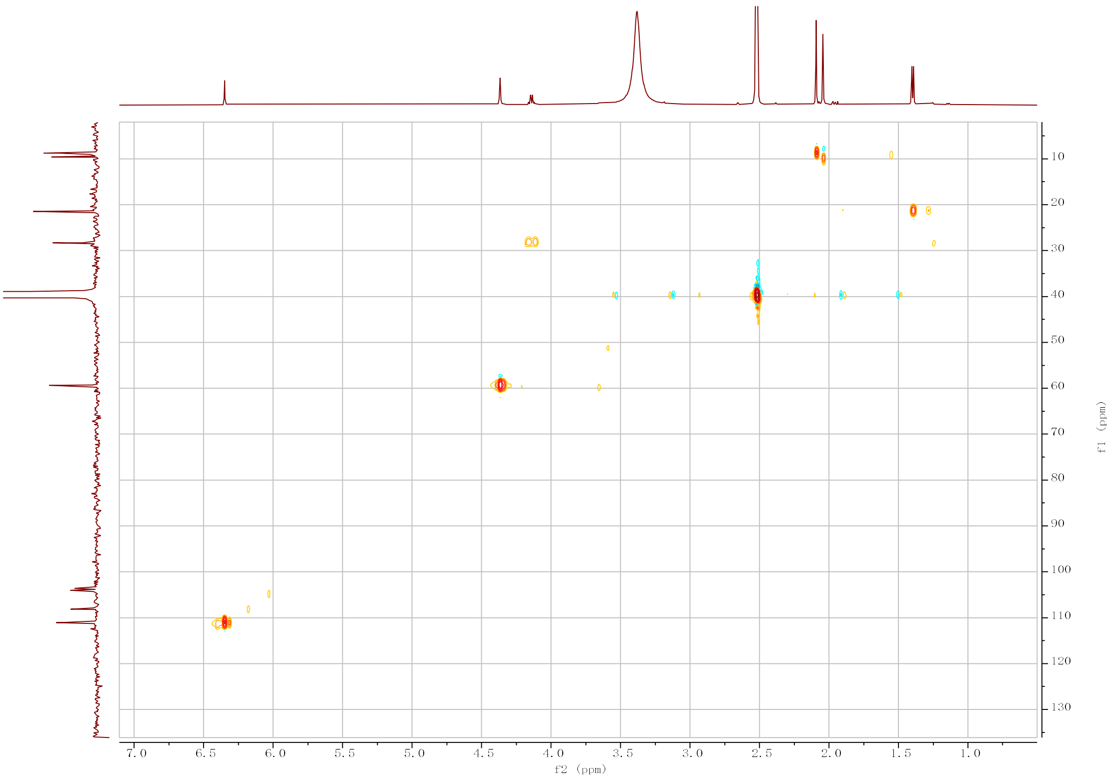

Figure S11. The HSQC Spectrum of compound 2 in DMSO-*d*₆.

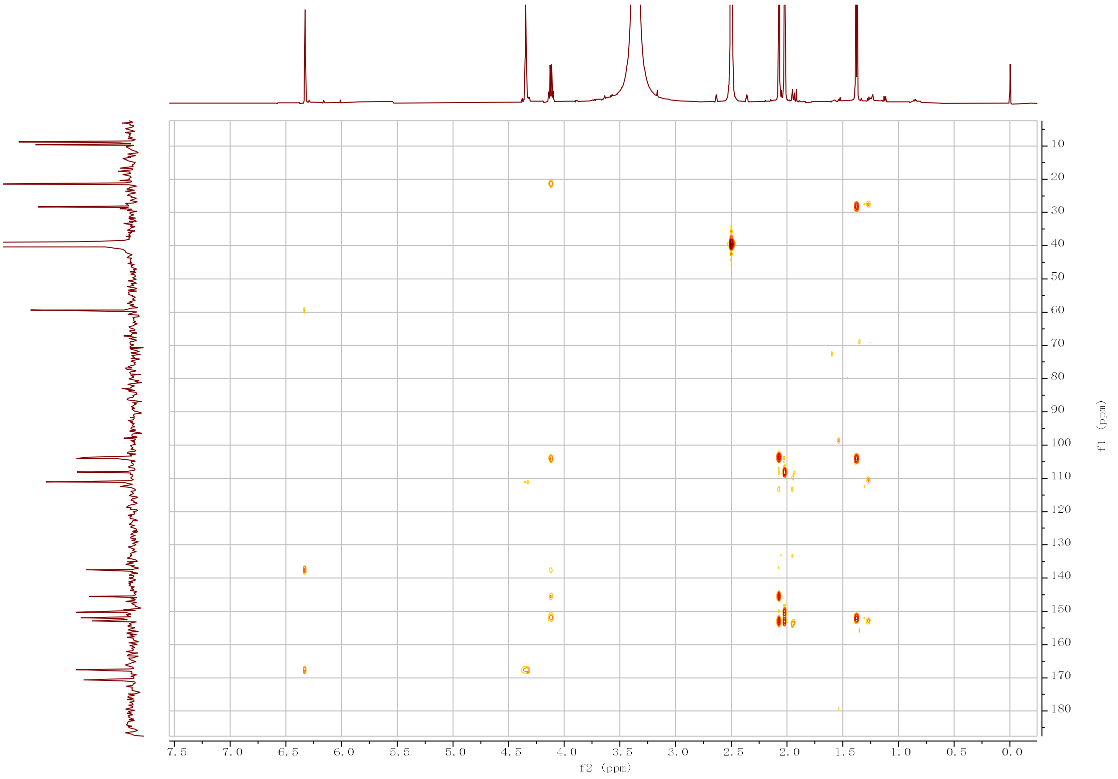

Figure S12. The HMBC Spectrum of compound 2 in DMSO-*d*₆.

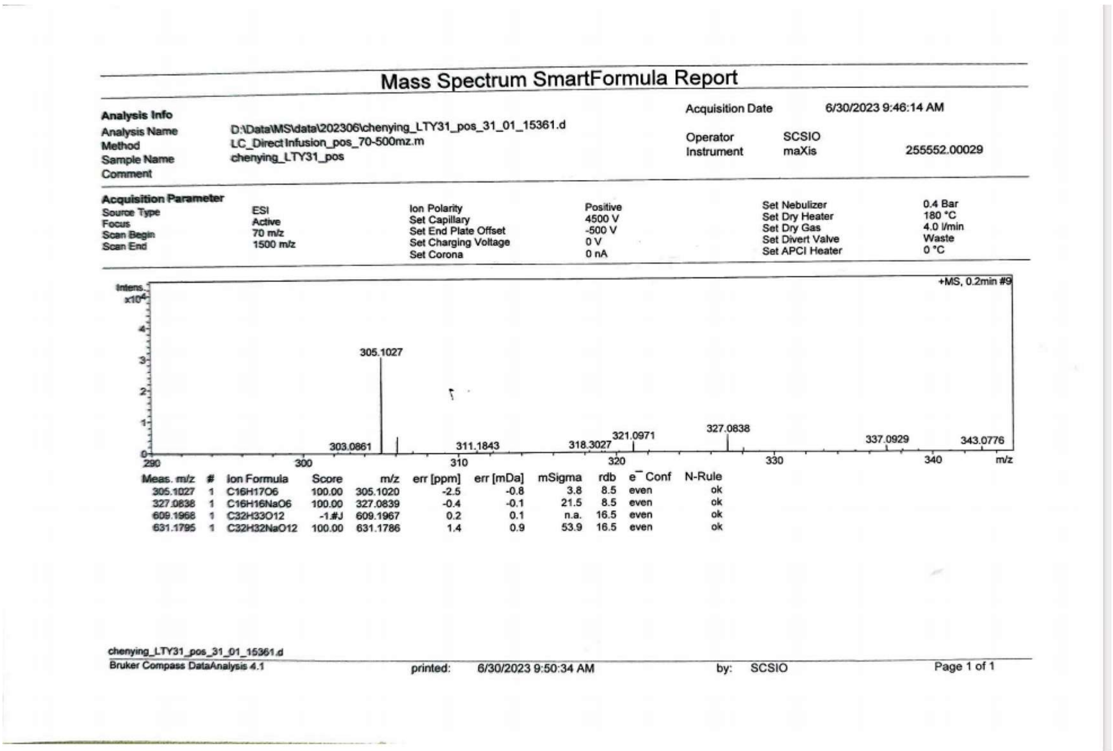

Mass Spectrum SmartFormula Report

Analysis Info		Acquisition Date	6/30/2023 9:46:14 AM
Analysis Name	D:\Data\MS\data\202306\chenying_LTY31_pos_31_01_15361.d	Operator	SCSIO
Method	LC_Direct Infusion_pos_70-500mz.m	Instrument	maXis 255552.00029
Sample Name	chenying_LTY31_pos		
Comment			

Acquisition Parameter					
Source Type	ESI	Ion Polarity	Positive	Set Nebulizer	0.4 Bar
Focus	Active	Set Capillary	4500 V	Set Dry Heater	180 °C
Scan Begin	70 m/z	Set End Plate Offset	-500 V	Set Dry Gas	4.0 l/min
Scan End	1500 m/z	Set Charging Voltage	0 V	Set Divert Valve	Waste
		Set Corona	0 nA	Set APCI Heater	0 °C

Meas. m/z	#	Ion Formula	Score	m/z	err [ppm]	err [mDa]	mSigma	rdb	e¯ Conf	N-Rule
305.1027	1	C16H17O6	100.00	305.1020	-2.5	-0.8	3.8	8.5	even	ok
327.0838	1	C16H16NaO6	100.00	327.0839	-0.4	-0.1	21.5	8.5	even	ok
609.1968	1	C32H33O12	-1.#J	609.1967	0.2	0.1	n.a.	16.5	even	ok
631.1795	1	C32H32NaO12	100.00	631.1786	1.4	0.9	53.9	16.5	even	ok

chenying_LTY31_pos_31_01_15361.d
Bruker Compass DataAnalysis 4.1 printed: 6/30/2023 9:50:34 AM by: SCSIO Page 1 of 1

Figure S13. The HRESIMS spectrum of compound 2 in MeOH.

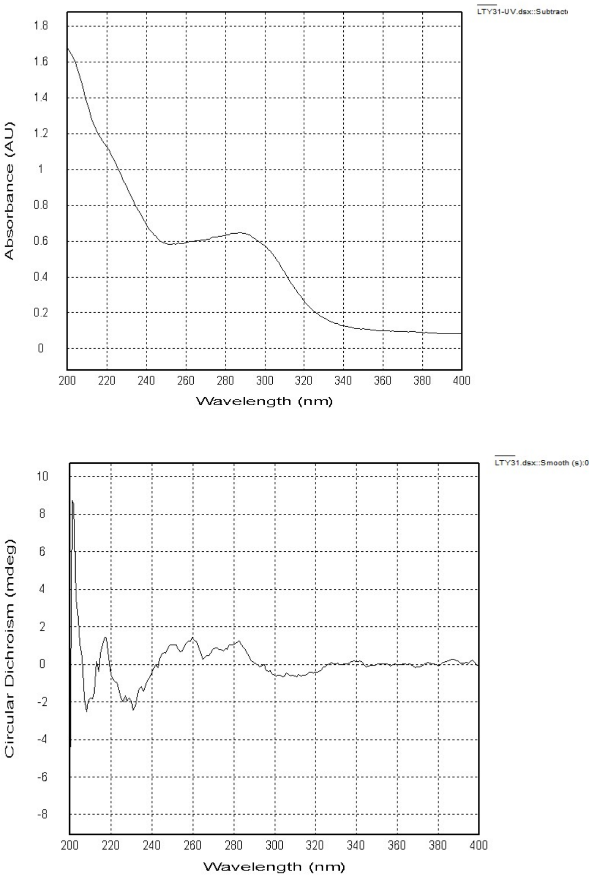

Figure S14. The UV and experimental ECD spectrum of compound 2.

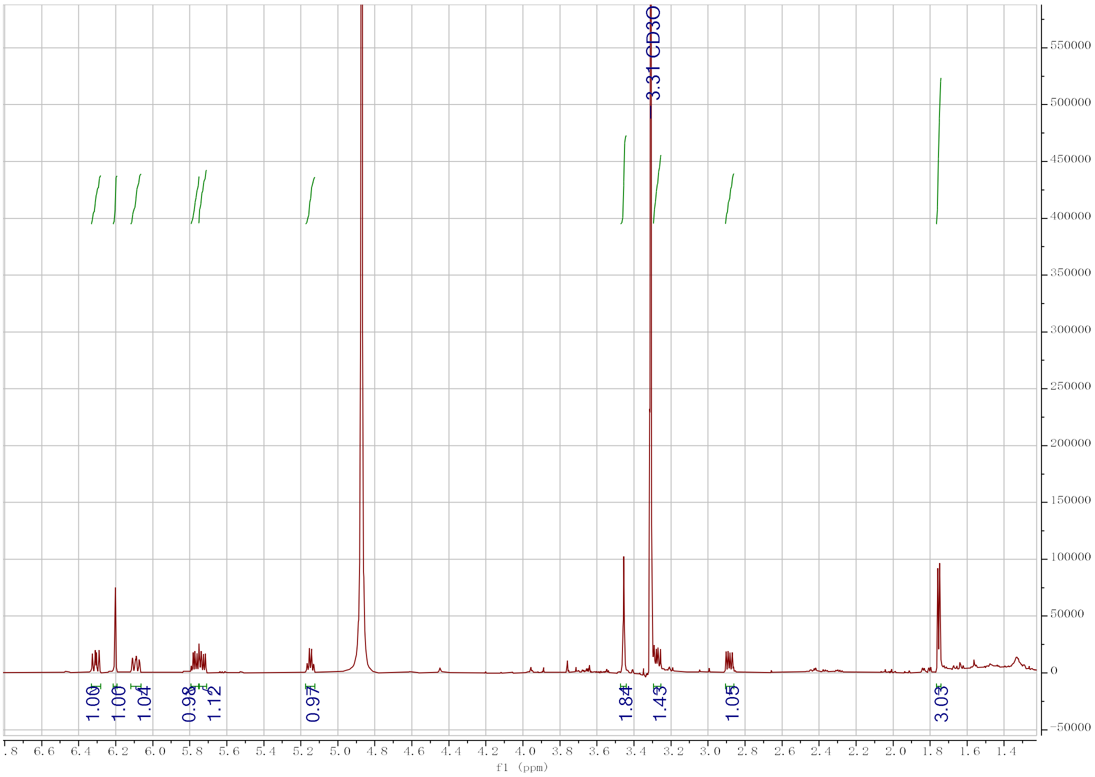

Figure S15. The ^{1}H NMR Spectrum of compound 3 in CD_3OD.

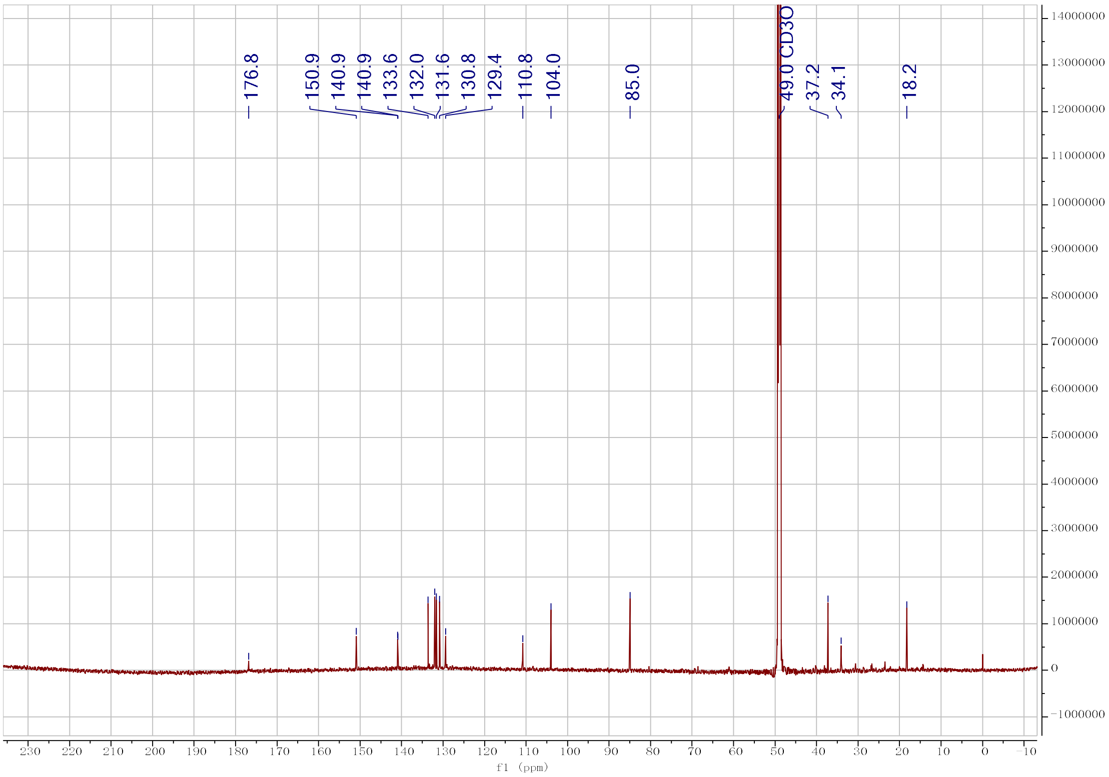

Figure S16. The ^{13}C NMR Spectrum of compound 3 in CD_3OD.

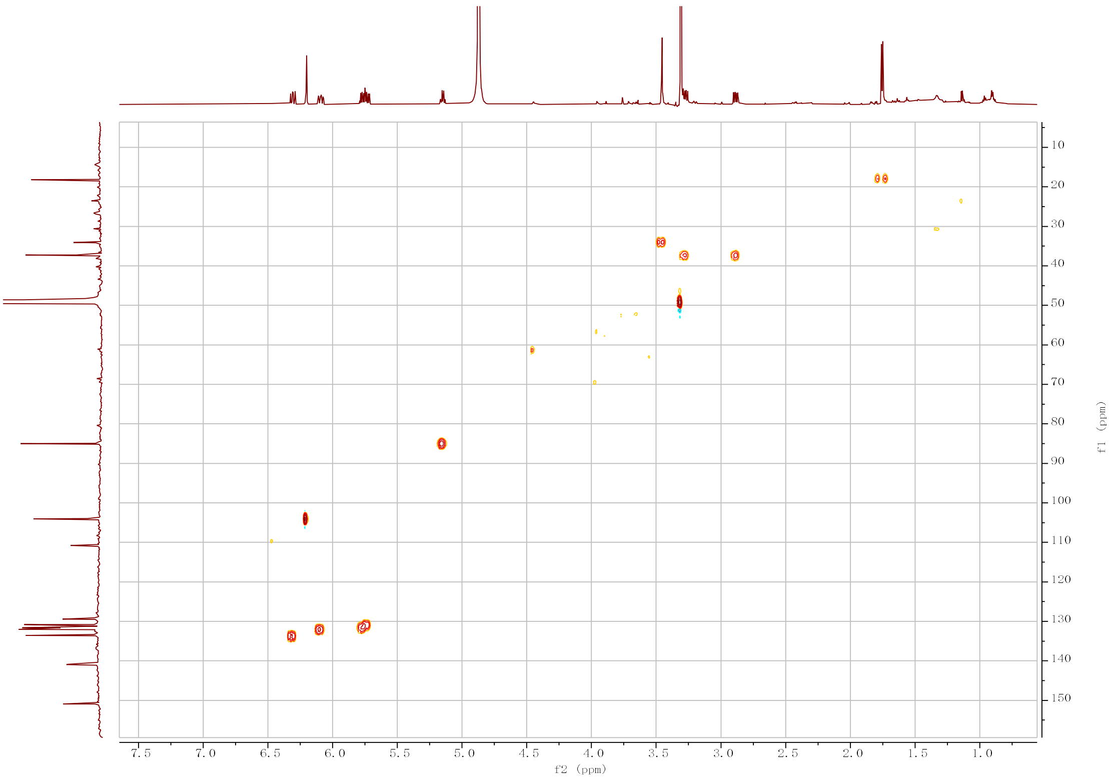

Figure S17. The HSQC Spectrum of compound 3 in CD_3OD.

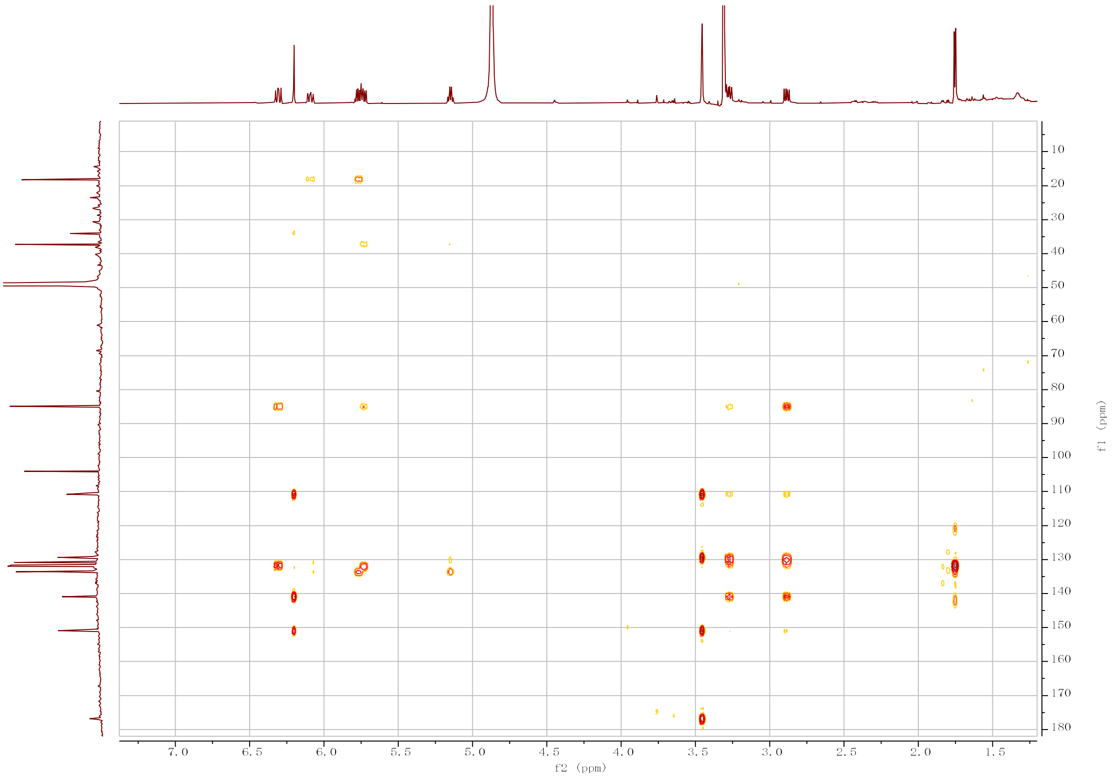

Figure S18. The HMBC Spectrum of compound 3 in CD_3OD.

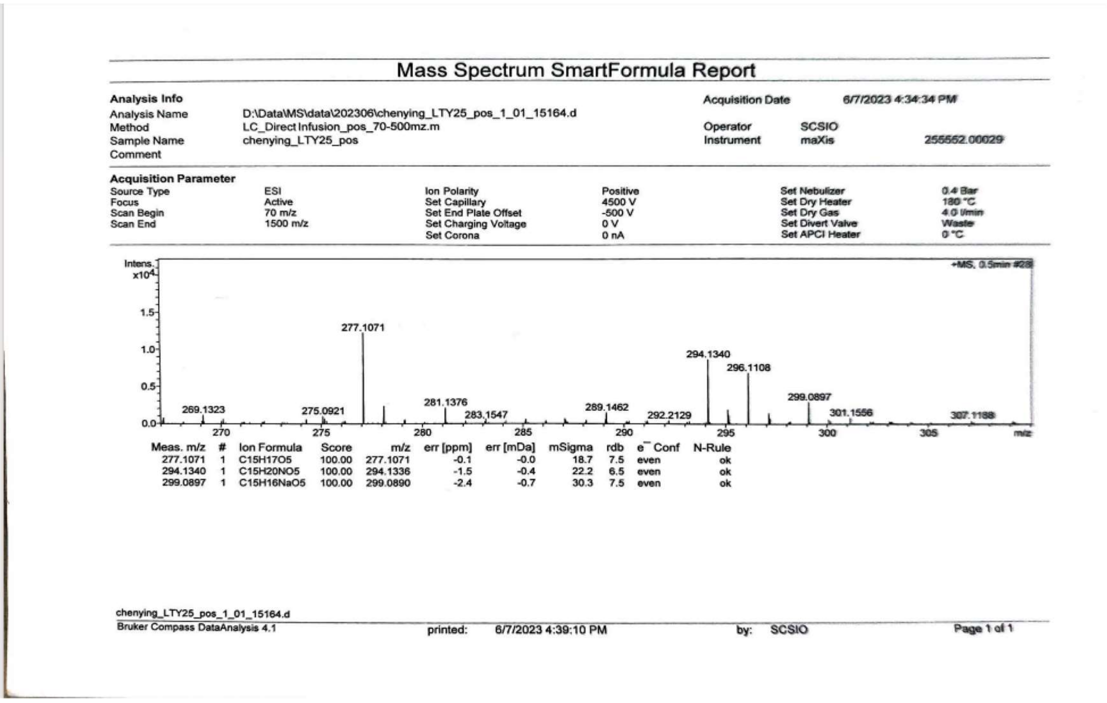

Figure S19. The HRESIMS spectrum of compound 3 in MeOH.

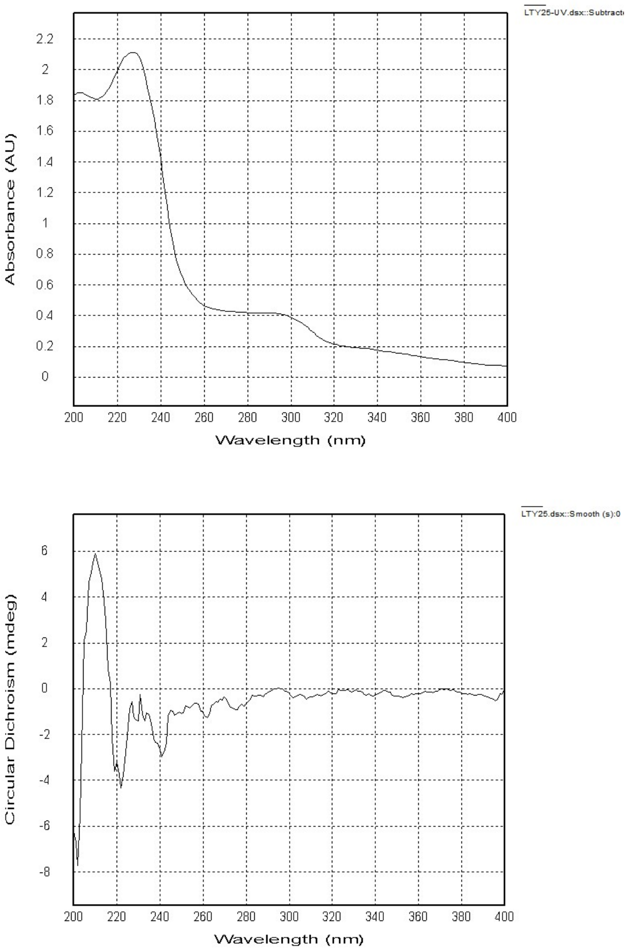

Figure S20. The UV and experimental ECD spectrum of compound 3.

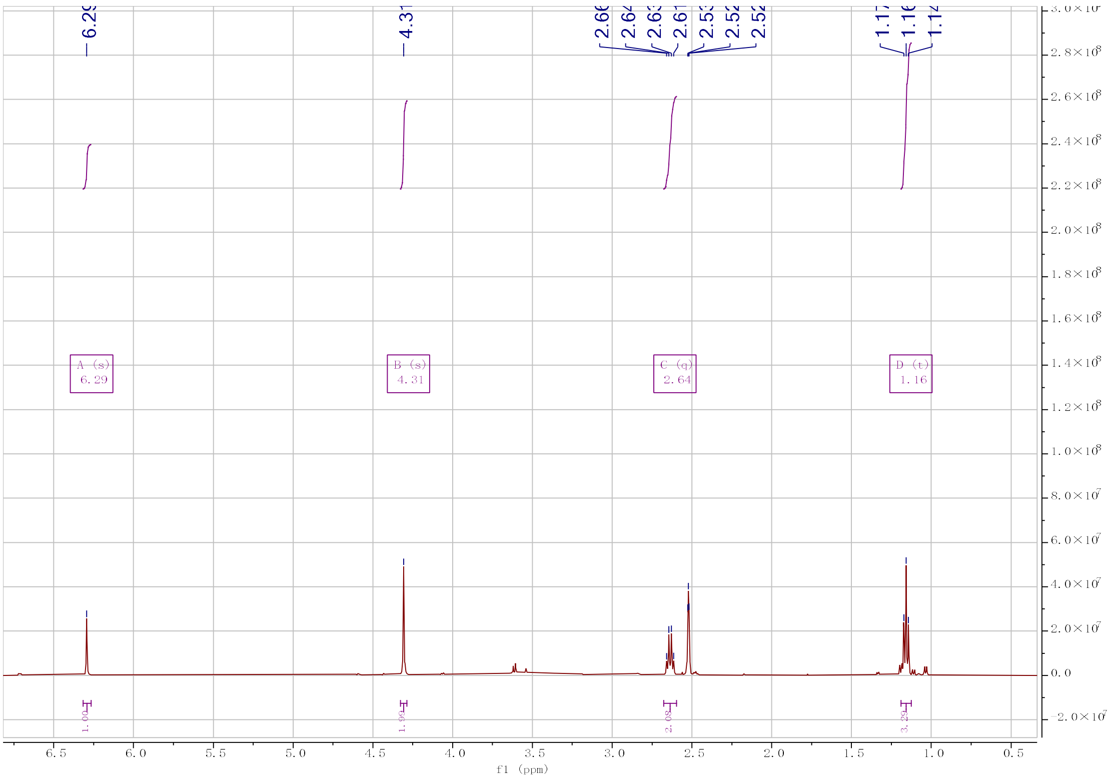

Figure S21. The ^{1}H NMR Spectrum of compound 4 in DMSO-d_6.

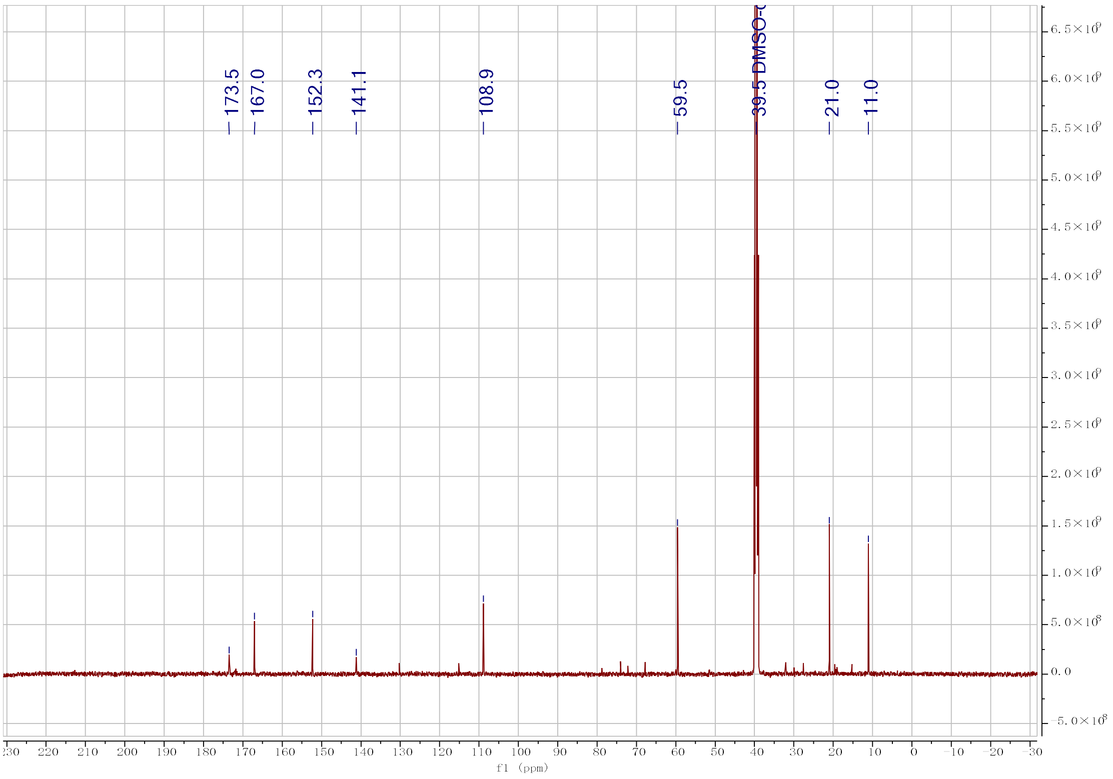

Figure S22. The ^{13}C NMR Spectrum of compound 4 in DMSO-d_6.

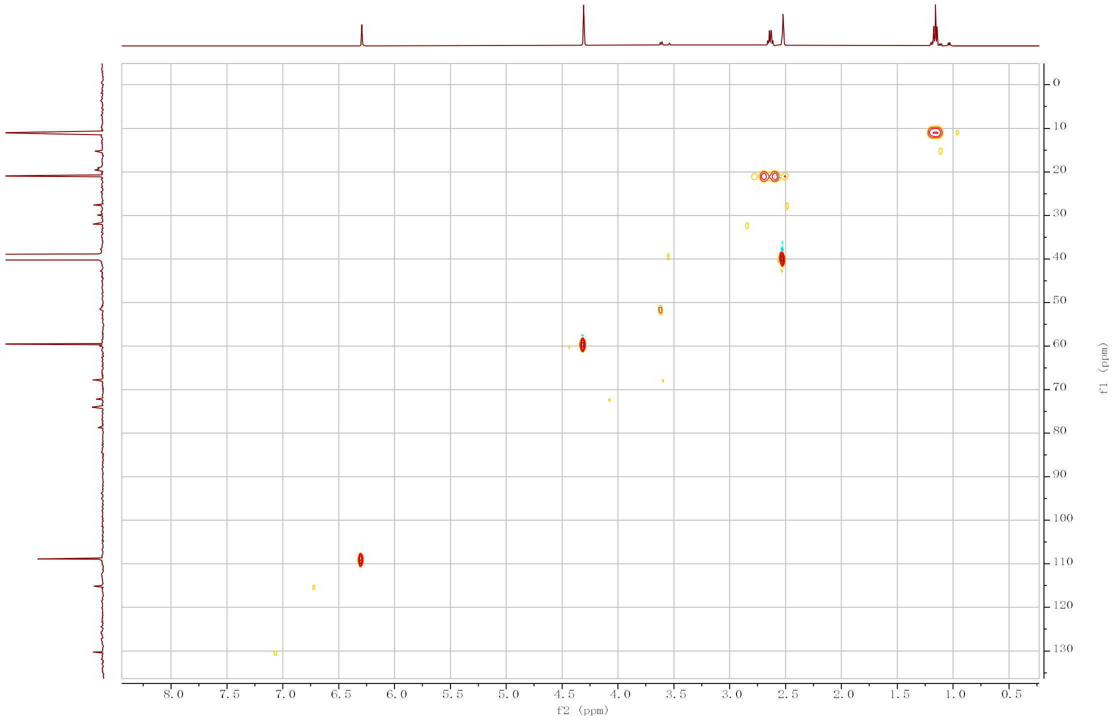

Figure S23. The HSQC Spectrum of compound 4 in DMSO-*d*6.

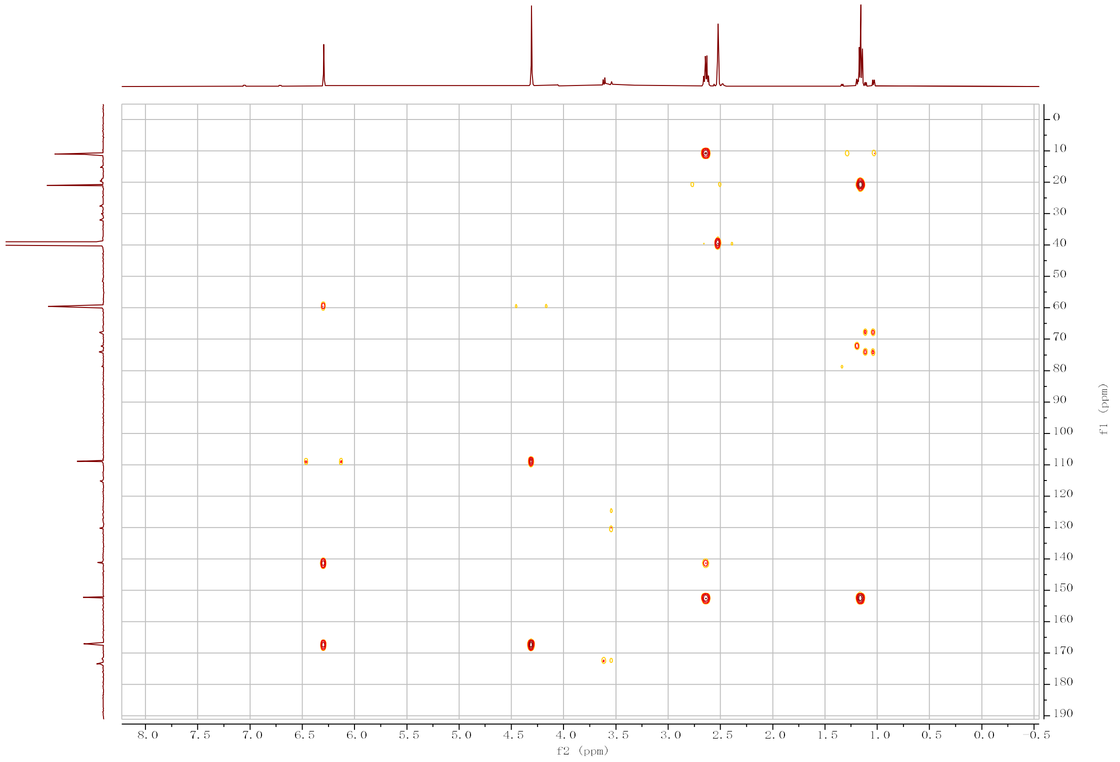

Figure S24. The HMBC Spectrum of compound 4 in DMSO-*d*6.

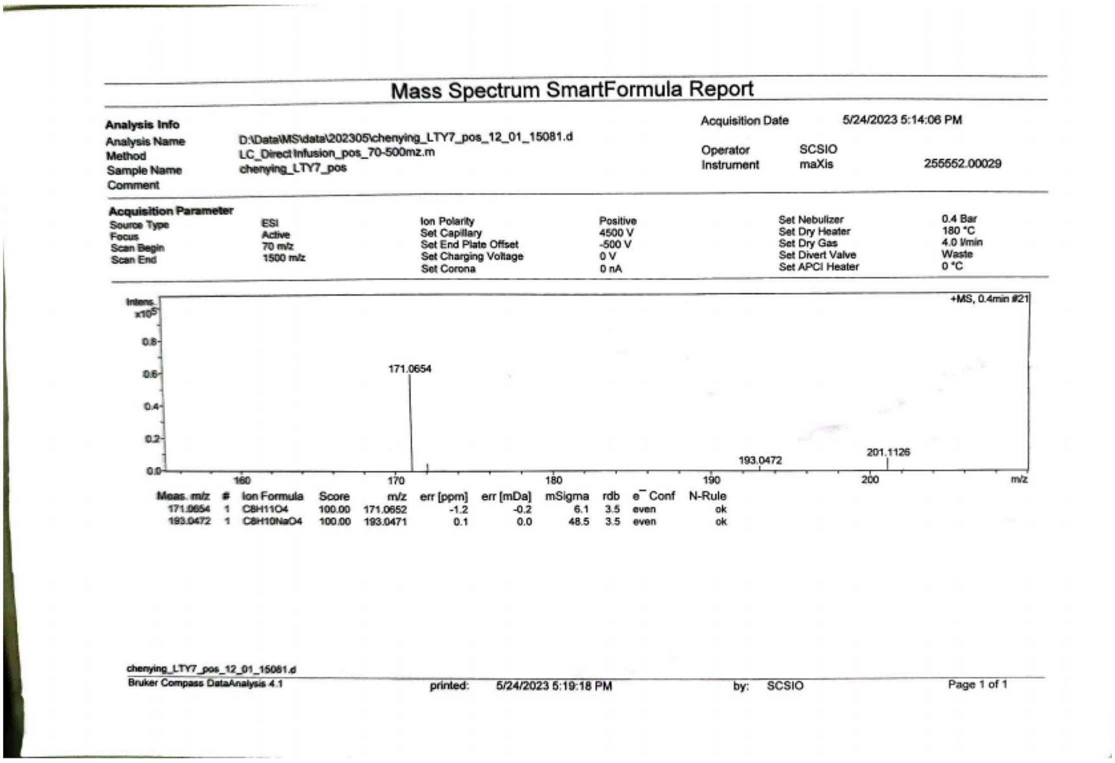

Figure S25. The HRESIMS spectrum of compound 4 in MeOH.

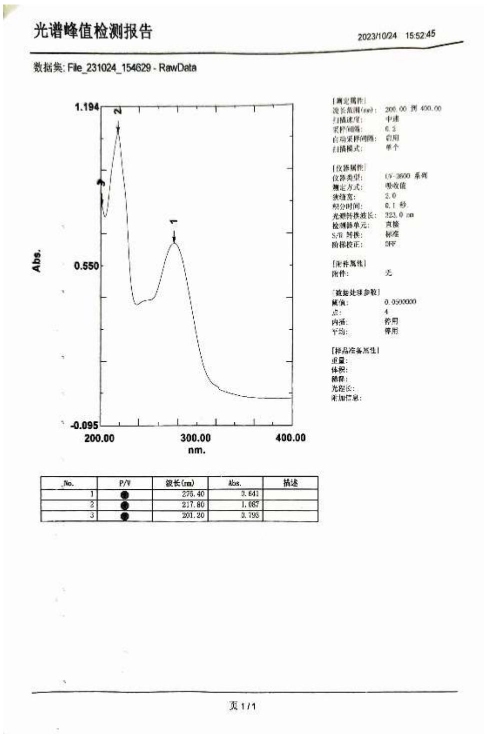

Figure S26. The UV spectrum of compound 4.

The physicochemical data of the known compounds 5~16.

kojicone (**5**): brown oil; ^{1}H NMR (500 MHz, CD$_3$OD, δ, ppm, *J*/Hz): δ_H 4.49 (s, 1H, H-22), 2.47 (m, 1H, H-16), 3.93 (q, *J* = 6.7 Hz, 1H, H-13), 1.79 (m, 1H, H-17), 1.52 (m, 1H, H-17), 1.66 (s,

3H, H-20), 1.72 (s, 3H, H-23), 1.38 (d, *J* = 6.7 Hz, 3H, H-21), 1.16 (d, *J* = 7.0 Hz, 3H, H-19), 0.97 (t, *J* = 7.4 Hz, 3H, H-18). ^{13}C NMR (125 MHz, CD_3OD, *δ*, ppm, *J*/Hz):200.5 (C, C-9), 192.2 (C, C-11), 177.0 (C, C-15), 173.0 (C, C-4), 171.1 (C, C-2), 164.7 (C, C-7), 155.3 (C, C-14), 138.4 (C, C-5), 113.3 (C, C-3), 112.7 (C, C-12), 83.5 (C, C-10), 73.3 (C, C-8), 60.9 (CH_2, C-22), 41.4 (CH, C-16), 29.6 (CH, C-13), 27.8 (CH_2, C-17), 23.6 (CH_3, C-20), 21.5 (CH_3, C-23), 20.6 (CH_3, C-21), 16.8 (CH_3, C-19), 11.7 (CH_3, C-18).

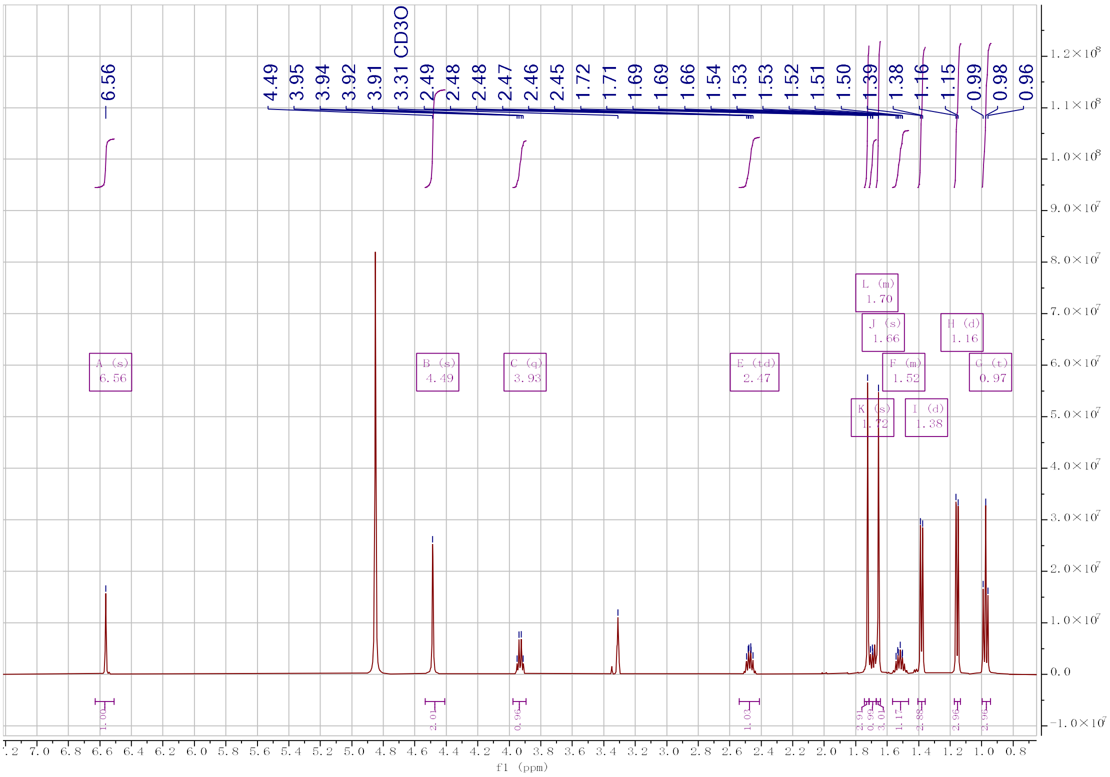

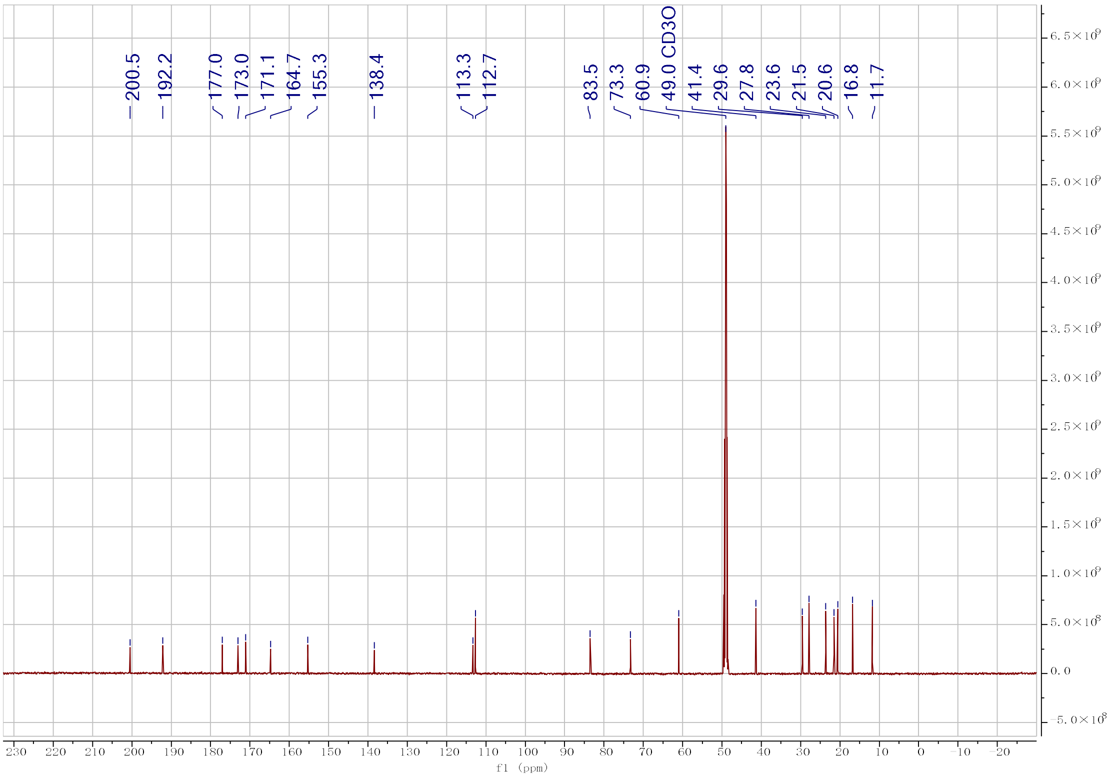

6,8-dihydroxy-3-((1*E*,3*E*)-penta-1,3-dien-1-yl)isochroman-1-one (**6**): brown oil; ^{1}H NMR (600 MHz, Methanol-d_4) δ 6.39 (dd, *J* = 15.3, 10.4 Hz, 1H, H-10), 6.24 (d, *J* = 2.2 Hz, 1H, H-5), 6.21 (d, *J* = 2.2 Hz, 1H, H-7), 6.10 (ddd, *J* = 14.9, 10.5, 2.0 Hz, 1H, H-11), 5.83 (dq, *J* = 13.9, 6.7 Hz, 1H, H-12), 5.69 (dd, *J* = 15.3, 6.8 Hz, 1H, H-9), 5.05 (m, 1H, H-3), 2.96 (dd, *J* = 7.0, 4.9 Hz, 2H, H-4), 1.77 (d, *J* = 6.8 Hz, 3H, H-13). ^{13}C NMR (126 MHz, MeOD) δ 171.4 (C, C-1), 166.4 (C, C-6), 165.6 (C, C-8), 143.1 (C, C-4a), 135.4 (CH, C-10), 133.1 (CH, C-12), 131.6 (CH, C-11), 127.6 (CH, C-9), 108.0 (CH, C-5), 102.3 (CH, C-7), 101.6 (C, C-8a), 80.7 (CH, C-3), 34.4 (CH_2, C-4), 18.2 (CH_3, C-13).

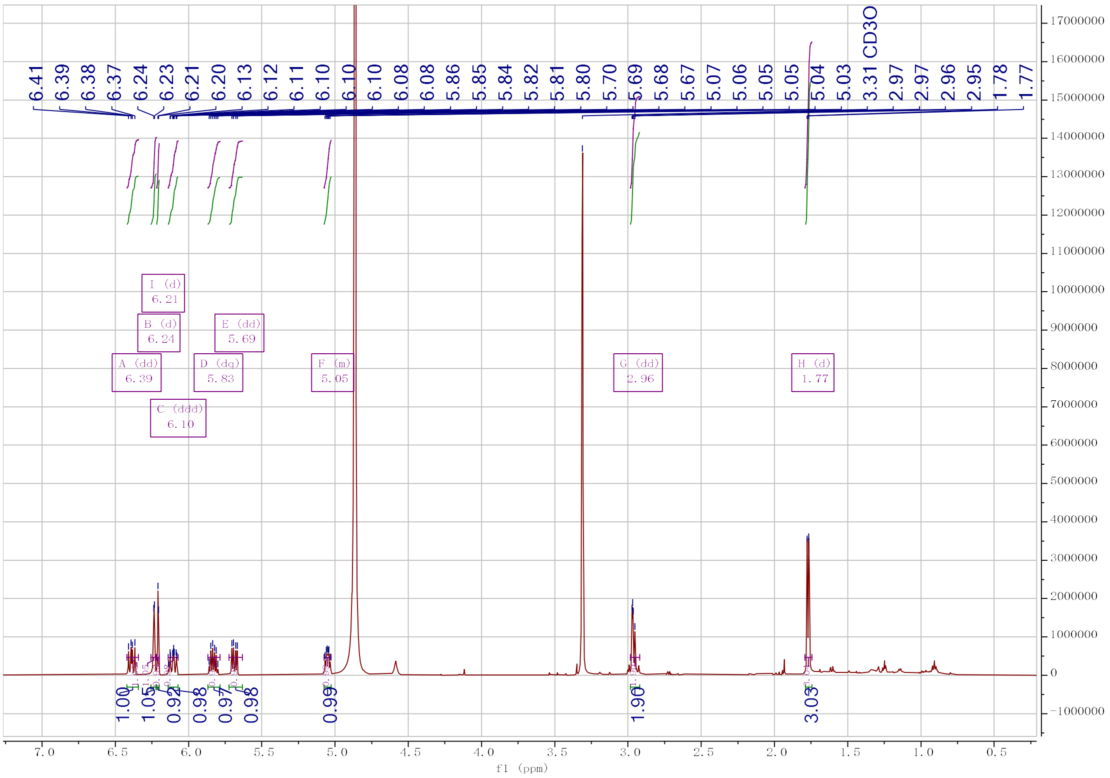

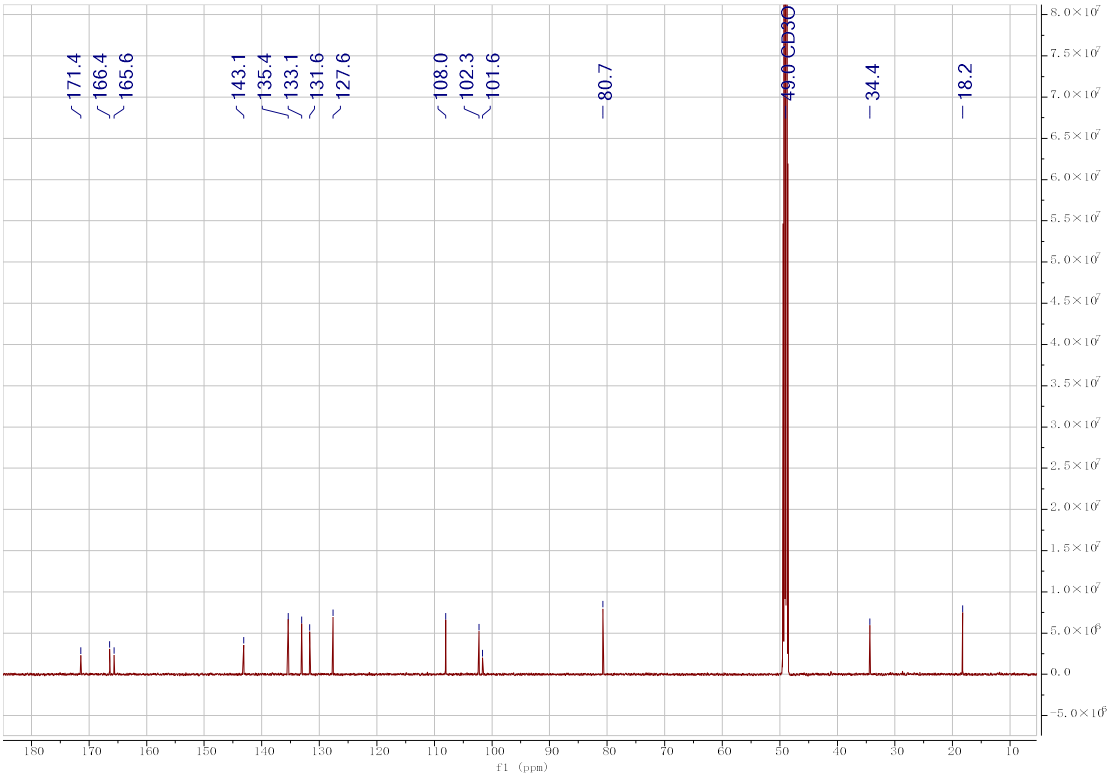

germicidin C (**7**): pale yellow solid; ^{1}H NMR (500 MHz, CD$_3$OD, δ, ppm, J/Hz): δ_H 5.98 (s, 1H, H-5), 2.48 (q, J = 7.0 Hz, 1H, H-7), 1.66 (m, 1H, H-8), 1.55 (m, 1H, H-8), 1.85 (s, 3H, H-9), 1.20 (d, J = 6.9 Hz, 3H, H-10), 0.89 (t, J = 7.4 Hz, 3H, H-9). ^{13}C NMR (125 MHz, CD$_3$OD, δ, ppm, J/Hz):169.2 (C, C-4), 168.2 (C, C-2), 168.2 (C, C-6), 100.4 (CH, C-5), 98.9 (C, C-3), 40.9 (CH, C-7), 28.5 (CH$_2$, C-8), 18.3 (CH$_3$, C-9), 11.9 (CH$_3$, C-10), 8.2 (CH$_3$, C-11).

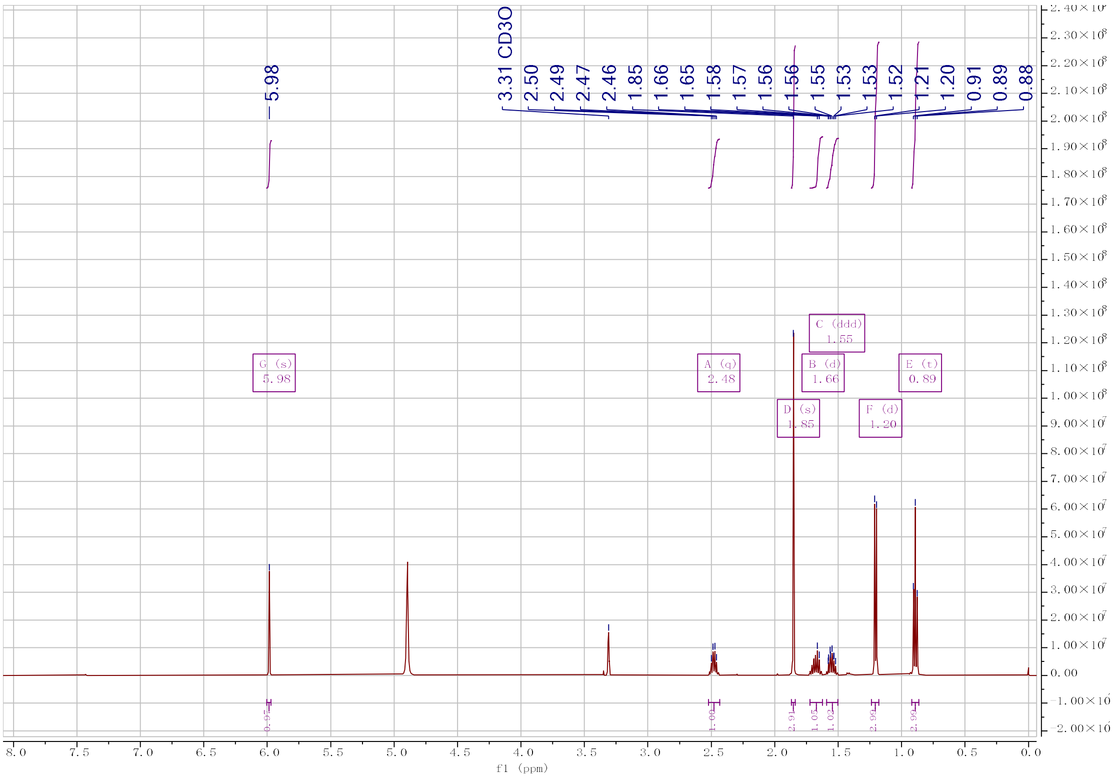

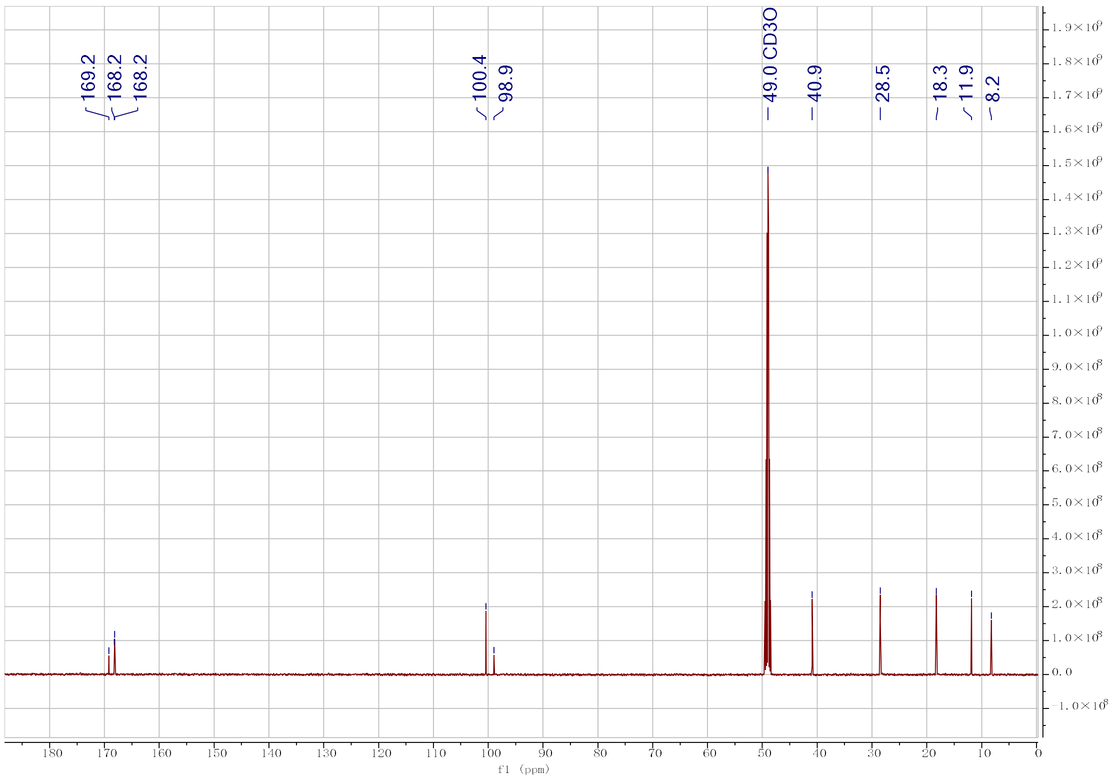

5-hydroxy-2-hydroxymethyl-4H-pyran-4-one (**8**): yellow solid; ^{1}H NMR (500 MHz, DMSO, *δ*, ppm, *J*/Hz): δ_H 8.00 (s, 1H, H-6), 6.34 (s, 1H, H-3), 4.29 (d, *J* = 5.5 Hz, 2H, H-1), 9.03 (s, 1H, 5-OH), 5.66 (s, 1H, 1-OH). ^{13}C NMR (125 MHz, DMSO, *δ*, ppm, *J*/Hz):174.1 (C, C-4), 168.3 (C, C-2), 145.9 (C, C-5), 139.6 (CH, C-6), 110.0 (CH, C-3), 59.7 (CH_2, C-1).

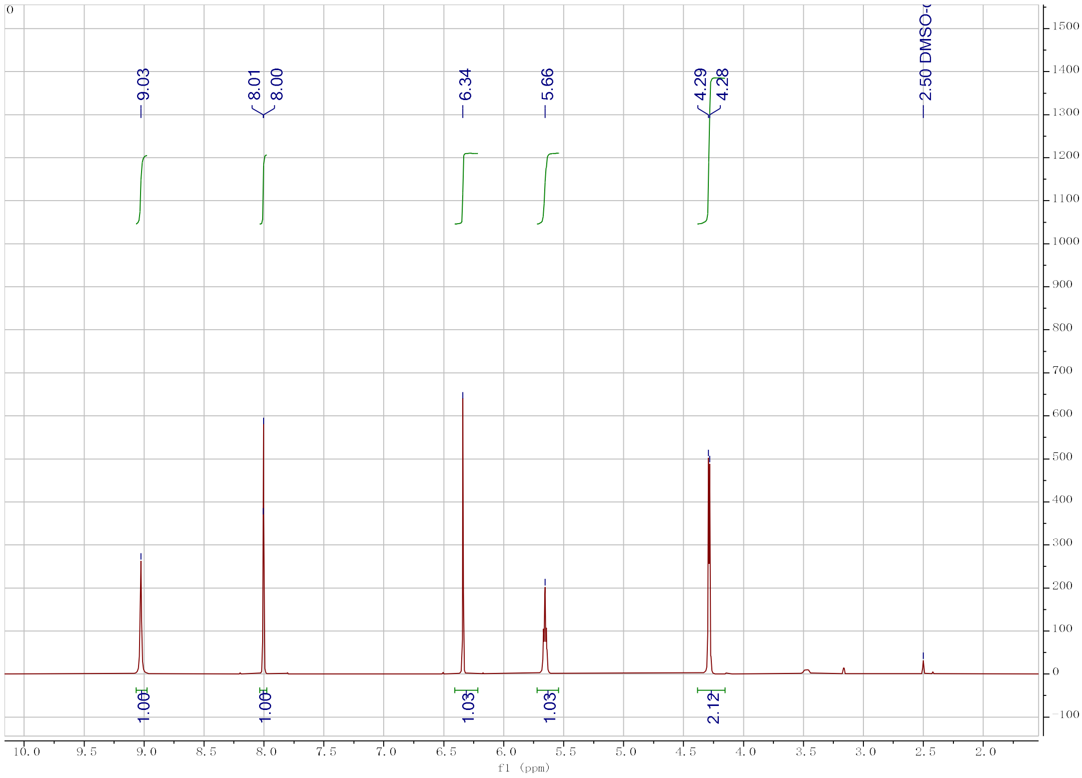

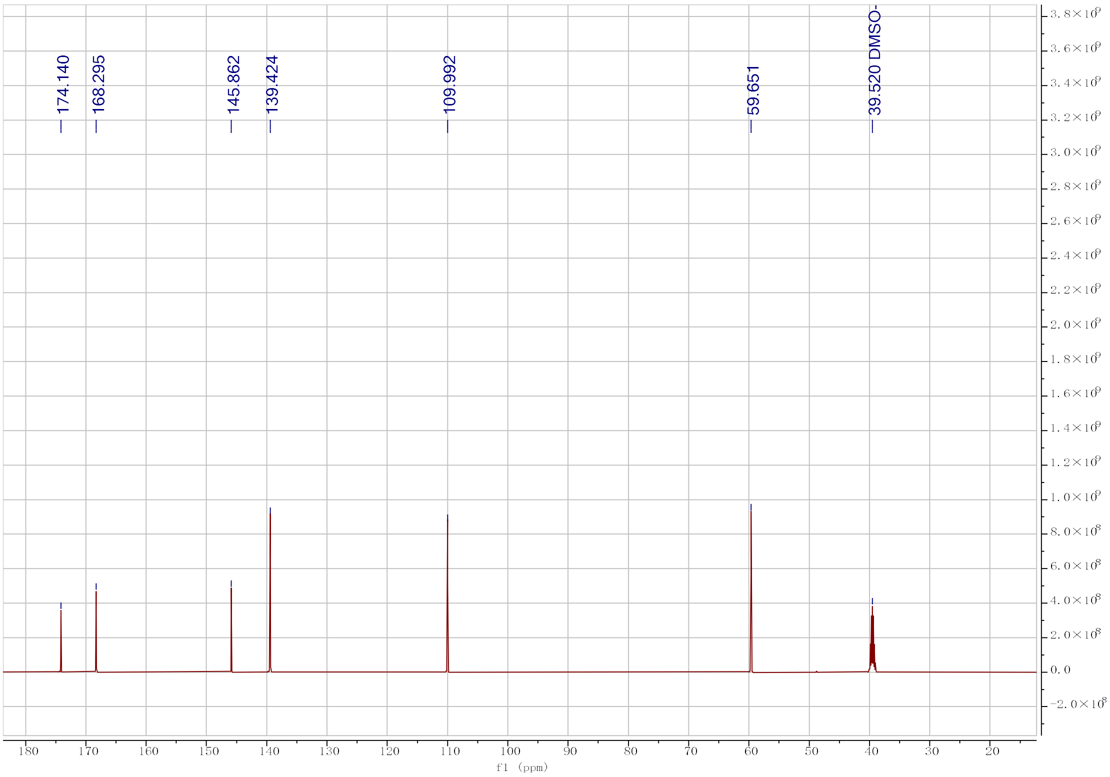

sydowione B (**9**): brown oil; ^{1}H NMR (500 MHz, DMSO, *δ*, ppm, *J*/Hz): δ_H 3.71 (m, 1H, H-8), 2.58 (dd, *J* = 14.0, 8.8 Hz 1H, H-7), 2.44 (dd, *J* = 14.1, 4.5 Hz 1H, H-7), 1.32 (m, 1H, H-9), 1.46 (ddd, *J* = 13.1, 7.5, 5.5Hz 1H, H-10), 1.16 (dt, *J* = 13.1, 7.6,Hz 1H, H-10), 0.87 (m, 3H, H-12), 0.86 (m, 3H, H-13), 1.88 (s, 3H, H-12), 1.82 (s, 3H, H-13). ^{13}C NMR (125 MHz, DMSO, *δ*, ppm, *J*/Hz):165.8 (C, C-5), 164.7 (C, C-4), 156.2 (C, C-6), 108.3 (C, C-5), 96.6 (C, C-3), 71.0 (CH, C-8), 39.0 (CH, C-9), 36.1 (CH_2, C-7), 25.6 (CH_2, C-10), 13.5 (CH_3, C-12), 11.8 (CH_3, C-11), 10.3 (CH_3, C-13), 9.3 (CH_3, C-14).

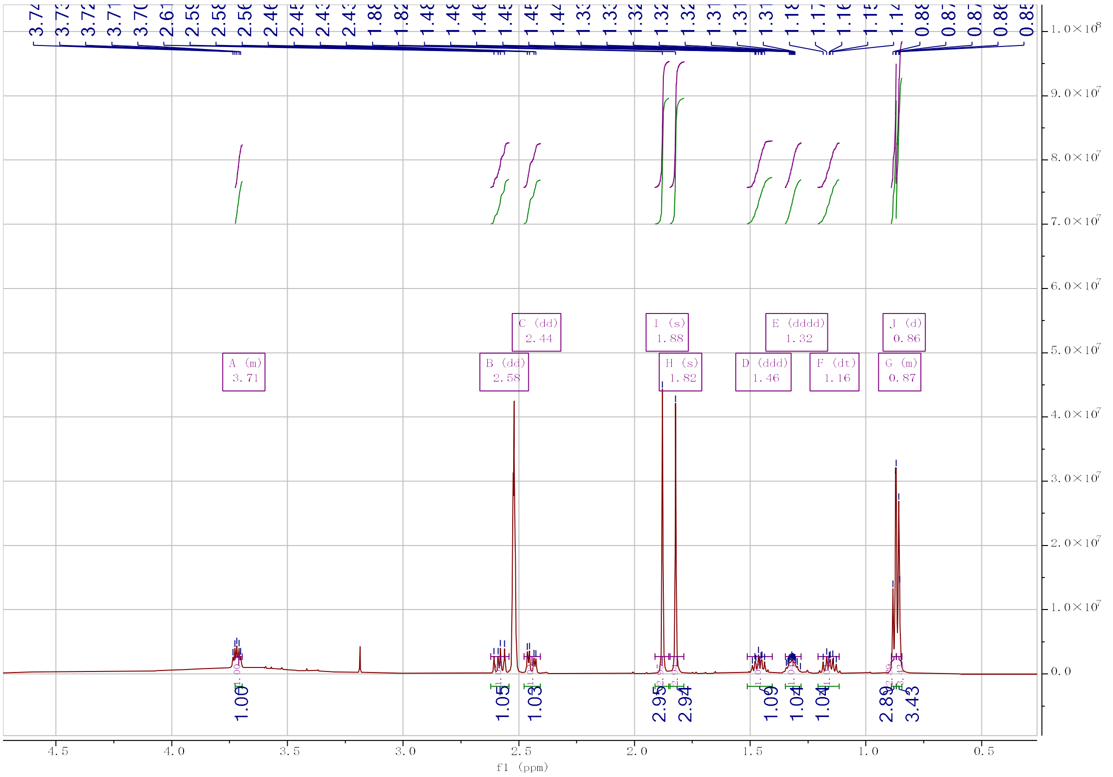

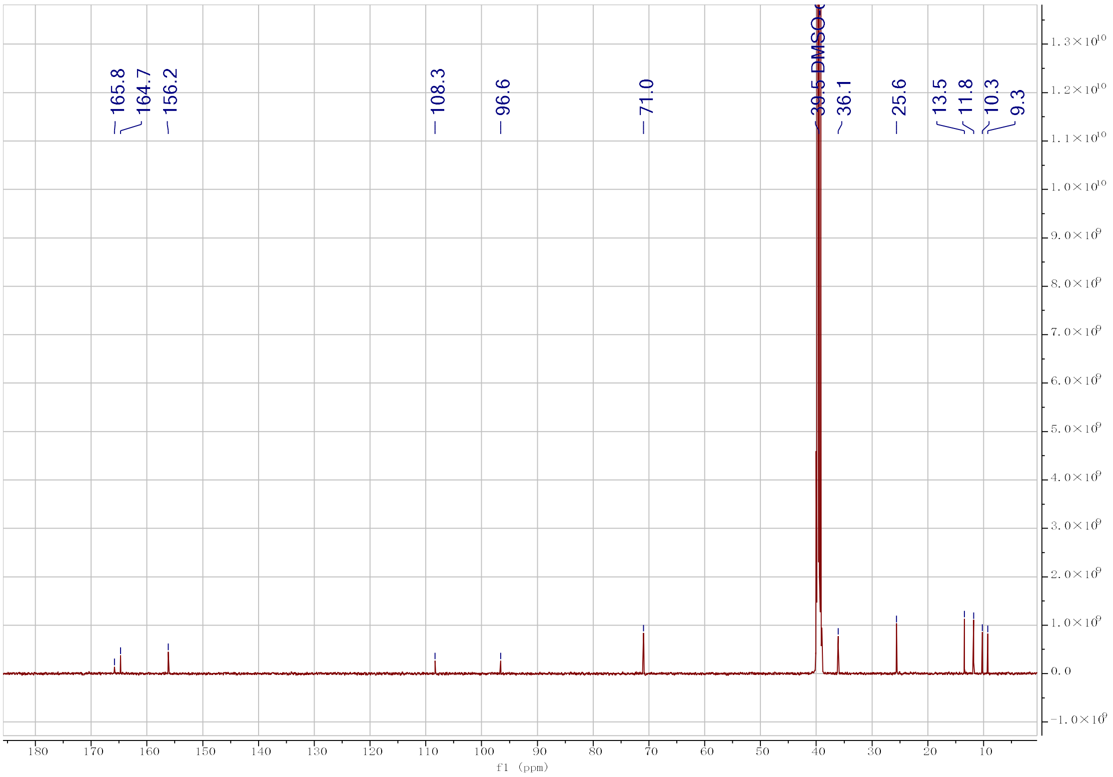

paecilpyrone A (**10**): brown oil; ^{1}H NMR (500 MHz, CD_3OD, δ, ppm, *J*/Hz): δ_H 2.68 (q, *J* = 6.9 Hz 1H, H-9), 1.76 (dq, *J* = 14.5, 7.2 Hz, 1H, H-10), 1.47 (dp, , *J* = 14.2, 7.3 Hz, 1H, H-10), 1.14 (d, *J* = 6.9 Hz, 3H, H-12), 0.93 (t, *J* = 7.4 Hz 3H, H-11), 1.92 (s, 3H, H-14), 1.94 (s, 3H, H-13). ^{13}C NMR (125 MHz, CD_3OD, δ, ppm, *J*/Hz):210.6 (C, C-8), 169.3 (C, C-5), 168.5 (C, C-3), 153.3 (C, C-1), 112.8 (C, C-6), 99.3 (C, C-4), 49.5 (CH, C-9), 26.8 (CH_2, C-10), 16.1 (CH_3, C-12), 10.3 (CH_3, C-14), 9.0 (CH_3, C-13).

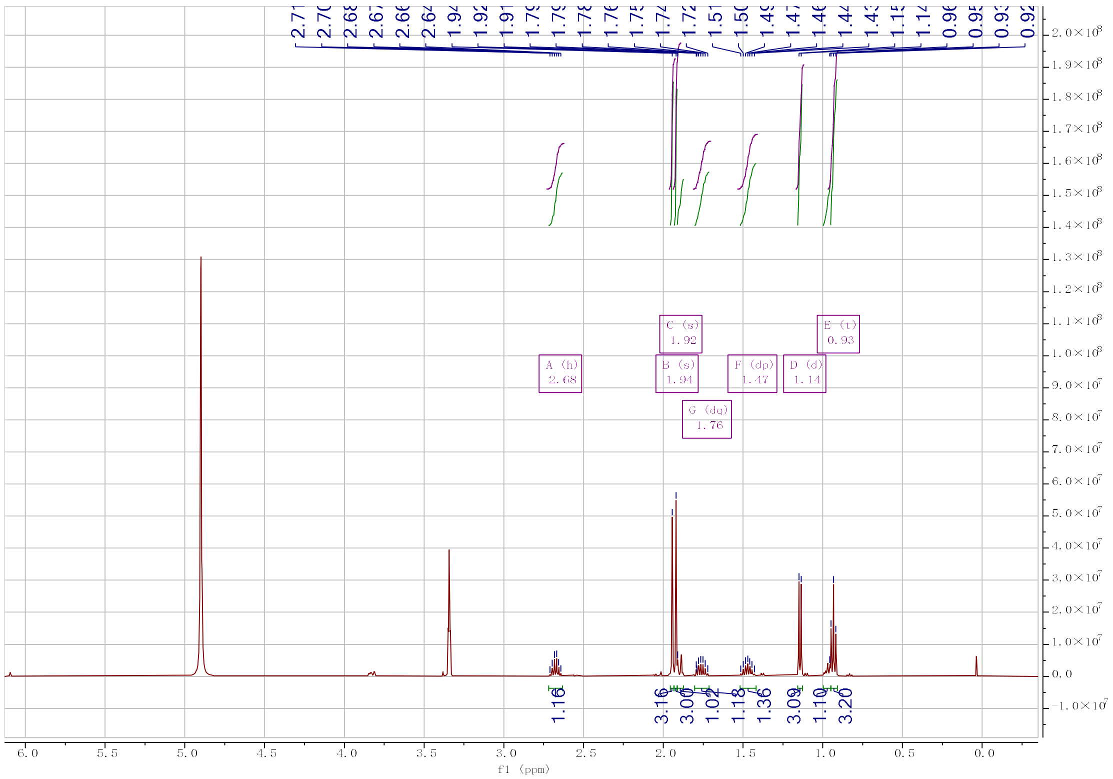

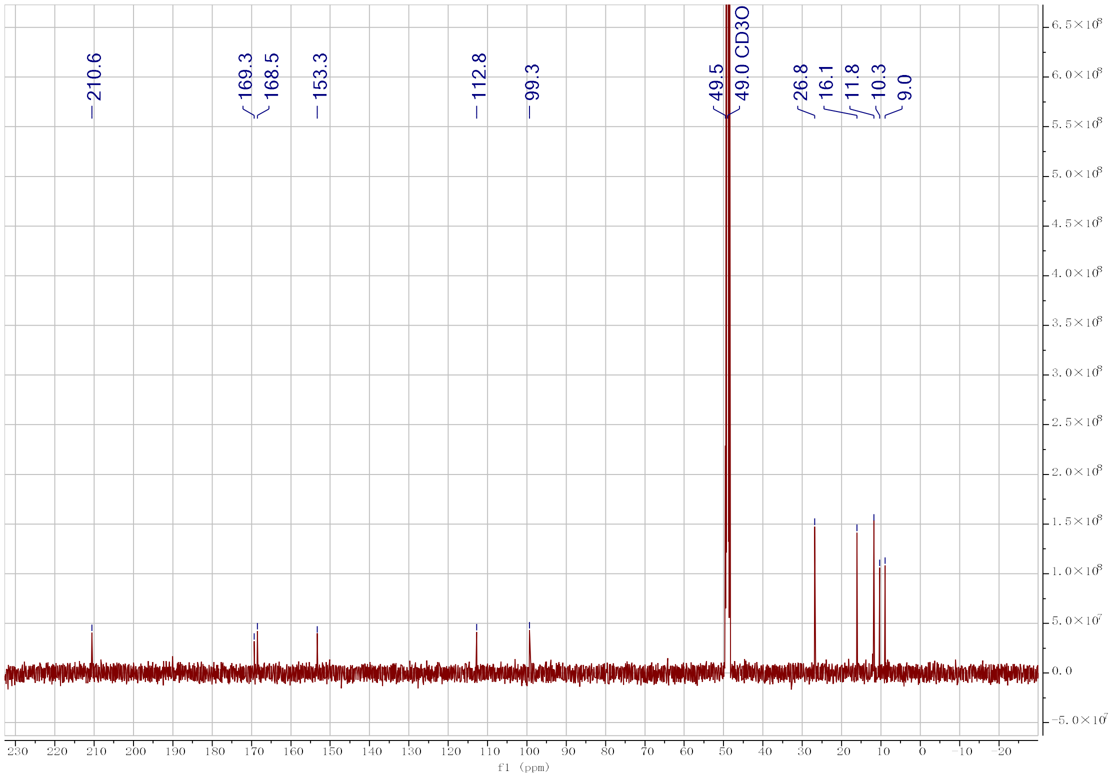

phomaligol A (**11**): yellow oil; ^{1}H NMR (500 MHz, Methanol-d_4) δ 5.61 (s, 1H, H-4), 3.89 (s, 3H, H-12), 2.45 (m, 1H, H-8), 1.70 (m, 1H, H-9), 1.64 (s, 3H, H-14), 1.56 (s, 3H, H-13), 1.50 (m, 1H, H-9), 1.13 (d, *J* = 7.0 Hz, 3H, H-11), 0.96 (t, *J* = 7.5 Hz, 3H, H-10). ^{13}C NMR (126 MHz, MeOD) δ 202.7 (C, C-1), 194.1 (C, C-5), 177.0 (C, C-7), 176.9 (C, C-3), 100.6 (CH, C-4), 83.3 (C, C-6) 74.1 (C, C-2), 57.7 (CH_3, C-12), 41.4 (CH, C-8), 27.9 (CH_2, C-9), 24.0 (CH_3, C-13), 22.2 (CH_3, C-14), 16.8 (CH_3, C-11), 11.7 (CH_3, C-10).

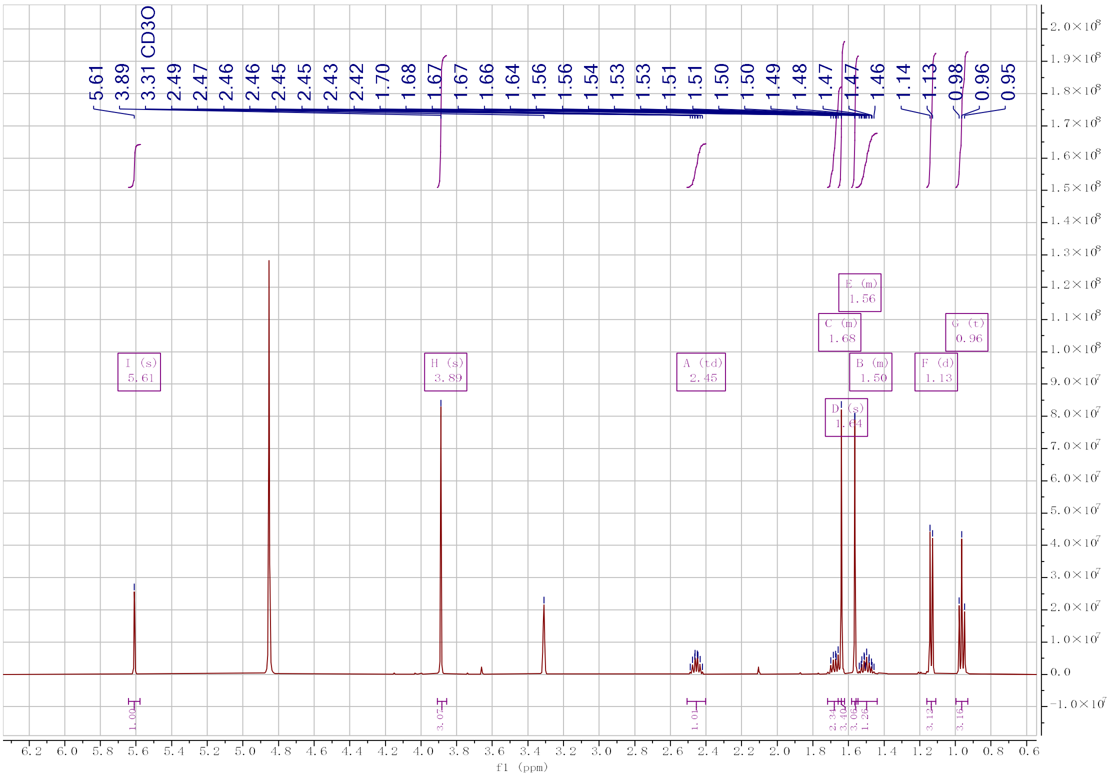

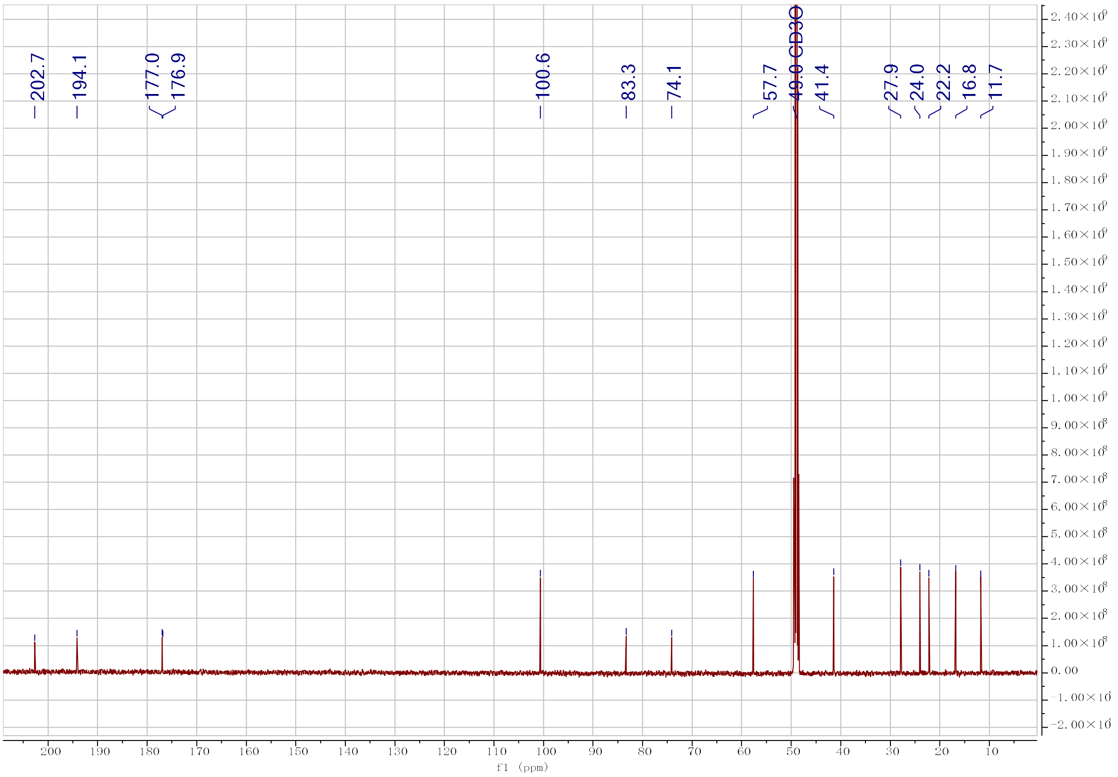

vanillic acid (**12**): white powder; ^{1}H NMR (500 MHz, Methanol-d_4) δ 7.55 (m, 2H, H-2, H-6), 6.84 (d, *J* = 8.6 Hz, 1H, H-5), 3.88 (d, *J* = 1.5 Hz, 3H, H-8). ^{13}C NMR (126 MHz, MeOD) δ 170.1 (C, C-7), 152.6 (C, C-4), 148.6 (C, C-3), 125.2 (CH, C-6), 123.1 (C, C-1), 115.8 (CH, C-5), 113.7 (CH, C-2), 56.3 (CH_3, C-8).

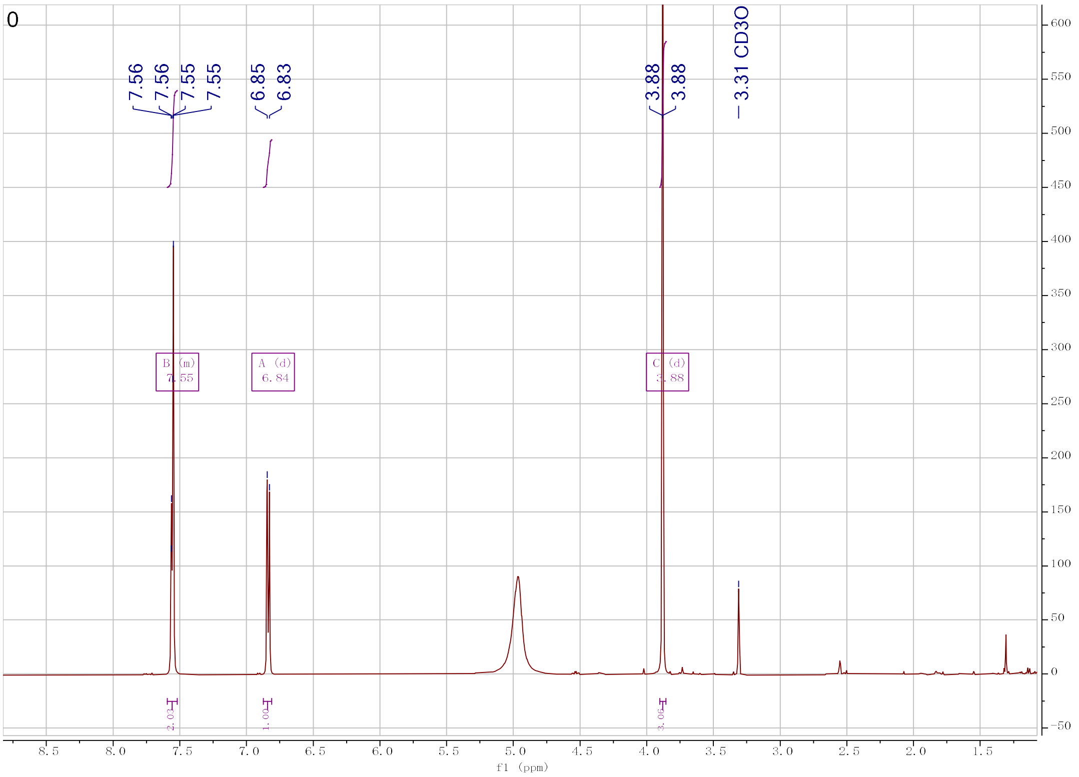

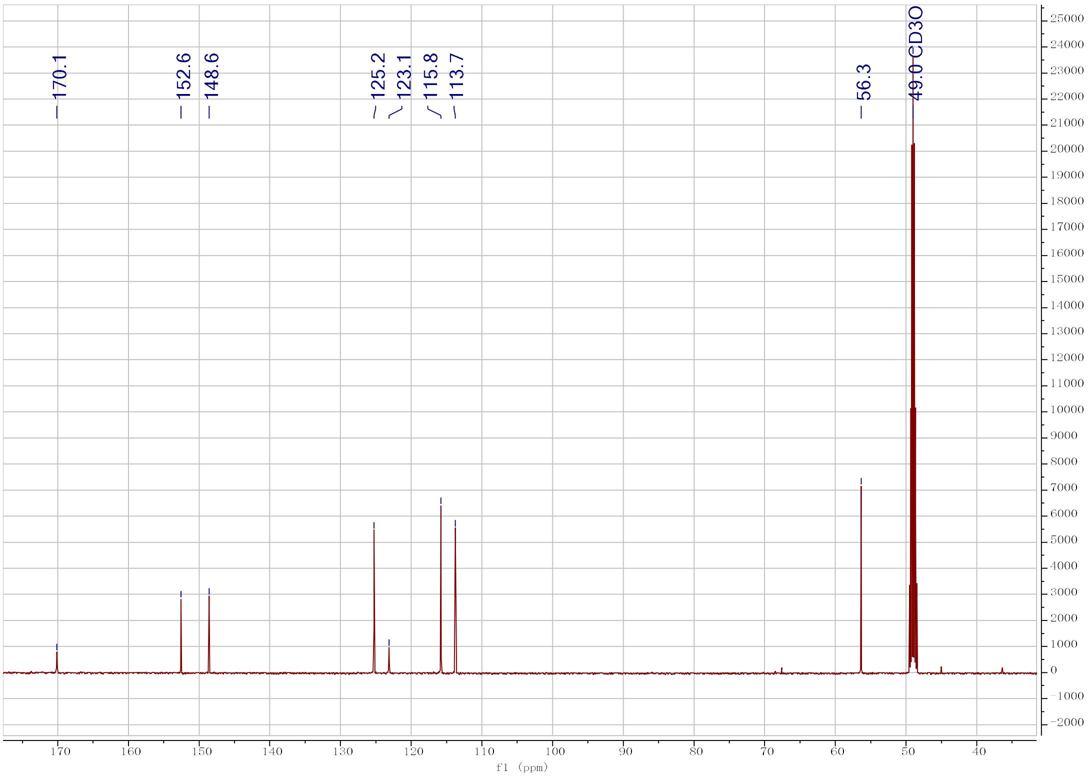

methyl-hydroxyphenylaceta (**13**): brown oil; ^{1}H NMR (500 MHz, Methanol-d_4) δ 7.14 (m, 1H, H-5′), 7.12 (m, 1H, H-2′) 6.78 (m, 1H, H-6′), 6.77 (m, 1H, H-4′),3.70 (s, 3H, 1-OCH_3), 3.64 (s, 2H, H-2). ^{13}C NMR (126 MHz, CD_3OD) δ 174.7 (C, C-1), 156.7 (C, C-3′), 132.1 (C, C-1′), 129.4 (CH, C-5′), 122.5 (CH, C-6′), 120.4 (CH, C-2′), 115.9 (CH, C-4′), 52.3 (CH_2, C-2), 36.4 (CH_3, 1-OCH_3).

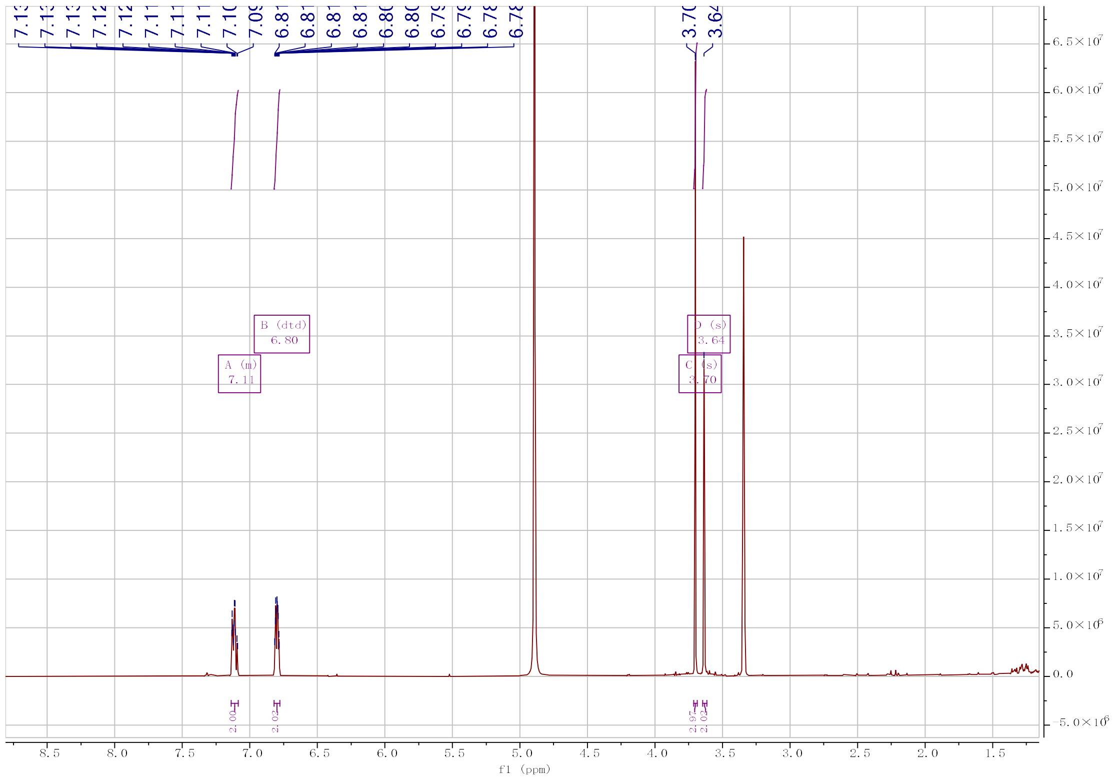

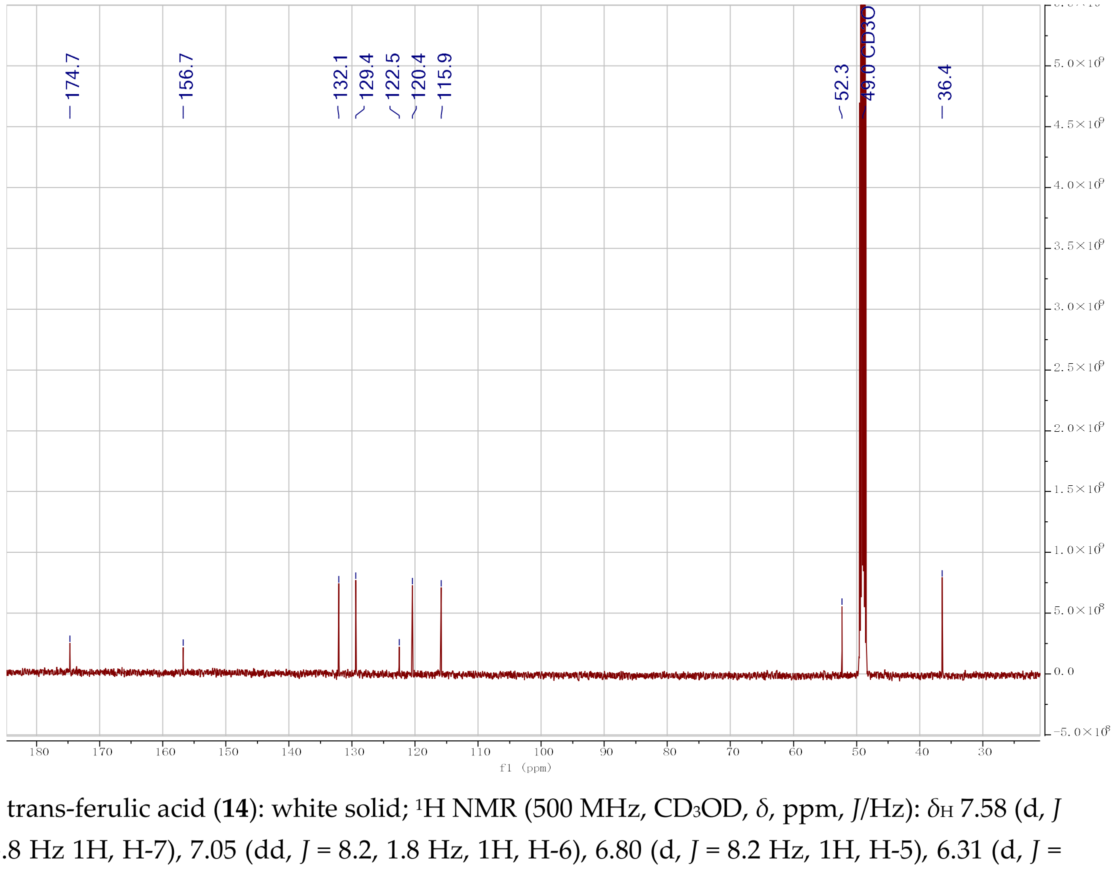

trans-ferulic acid (**14**): white solid; ^{1}H NMR (500 MHz, CD_3OD, δ, ppm, *J*/Hz): δ_H 7.58 (d, *J* = 15.8 Hz 1H, H-7), 7.05 (dd, *J* = 8.2, 1.8 Hz, 1H, H-6), 6.80 (d, *J* = 8.2 Hz, 1H, H-5), 6.31 (d, *J* = 15.8 Hz, 1H, H-8), 7.17 (d, *J* = 1.8 Hz 1H, H-2), 3.88 (s, 3H, 3-OMe). ^{13}C NMR (125 MHz, CD_3OD, δ, ppm, *J*/Hz):171.2 (C, C-9), 150.5 (C, C-4), 149.4 (C, C-3), 146.8 (CH, C-7), 127.8 (C, C-1), 124.0 (CH, C-6), 116.5 (CH, C-5), 116.1 (CH, C-8), 111.7 (CH, C-2), 56.4 (CH_3, 3-OMe).

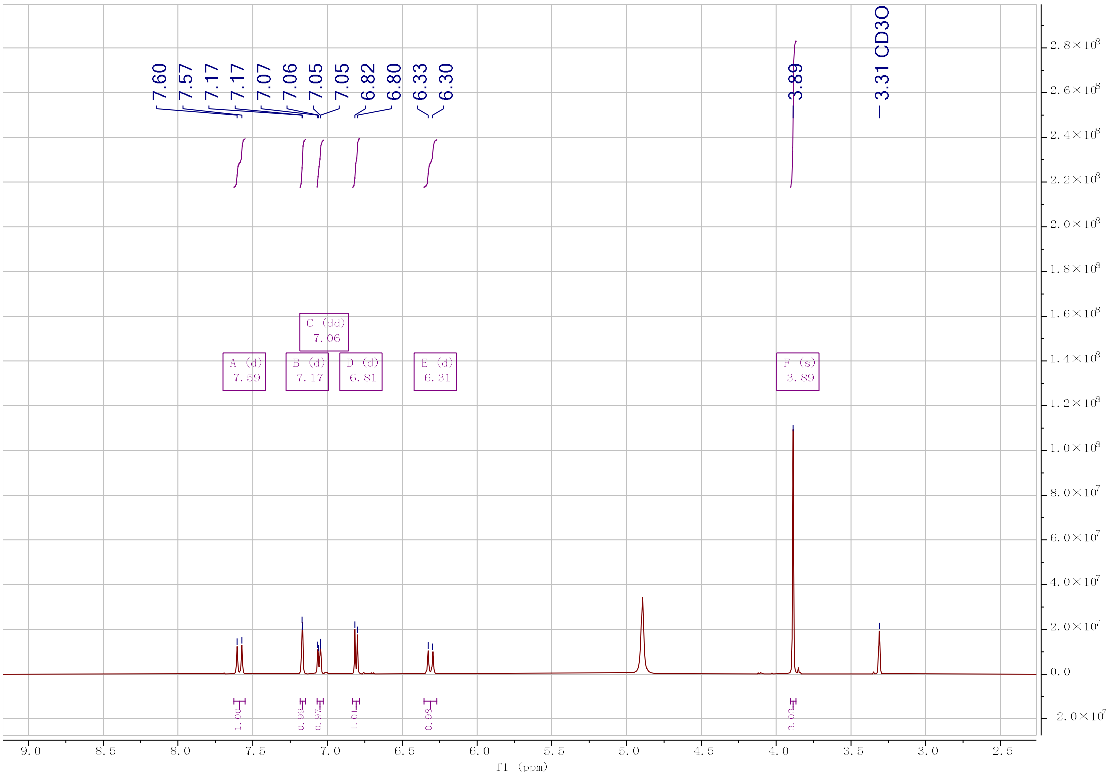

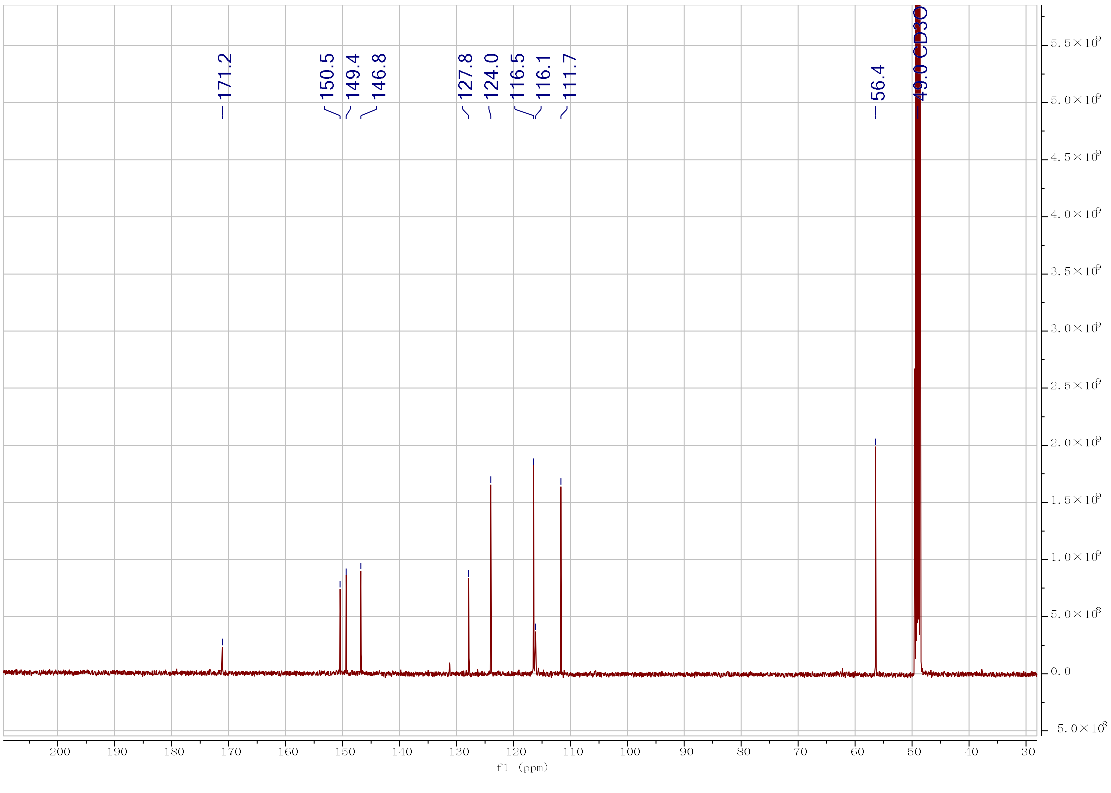

nicotinic acid (**15**): pale yellow powder; ^{1}H NMR (500 MHz, CD_3OD, δ, ppm, *J*/Hz): δ_H 9.14 (s, 1H, H-2), 8.40 (d, 1H, *J* = 7.5 Hz, H-5), 7.56 (dd, *J* = 8.0, 4.8 Hz, 1H, H-5), 8.70 (s, 1H, H-6). ^{13}C NMR (125 MHz, CD_3OD, δ, ppm, *J*/Hz):168.3 (C, COOH), 151.3 (C, C-2), 129.4 (C, C-3), 139.2 (C, C-4), 125.3 (C, C-5), 153.4 (C, C-6).

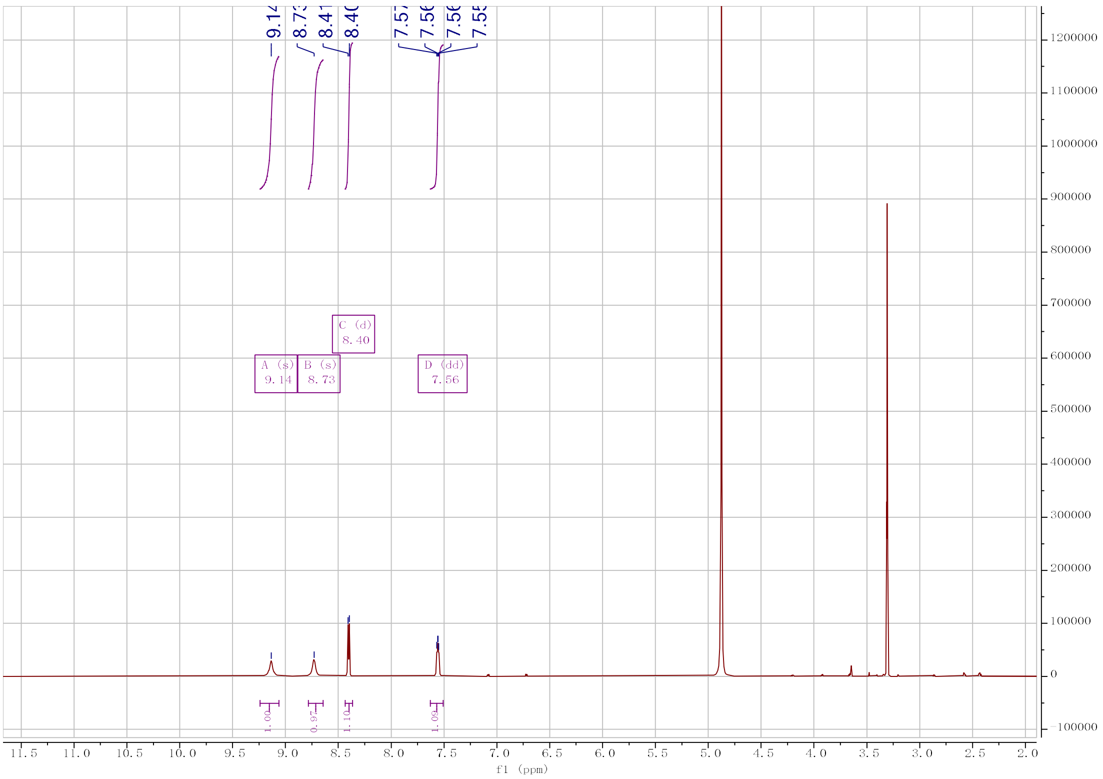

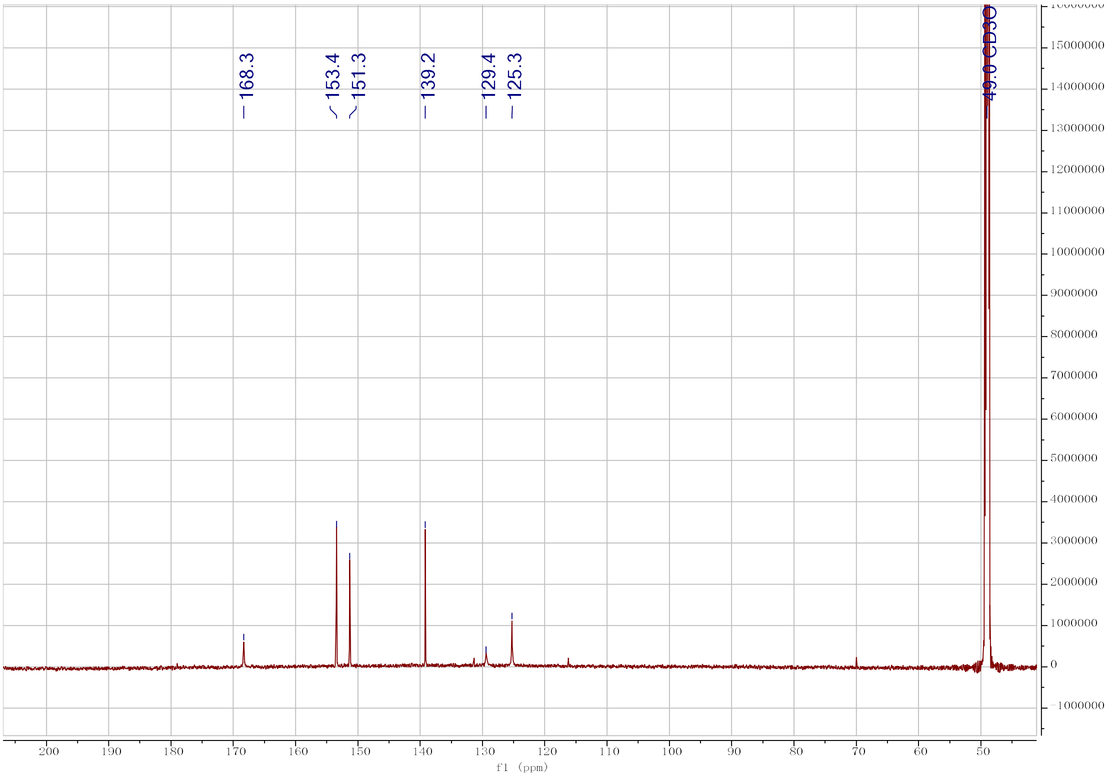

7,9-dihydroxy-3-(1H-indol-3-ylmethyl)-8-methoxy-2,3,11,11a-tetrahydro-6H-pyrazino[1,2-b]isoquinoline-1,4-dione (**16**): yellow oil; ^{1}H NMR (500 MHz, Methanol-d_4) δ 7.48 (d, *J* = 8.0 Hz, 1H, H-10), 7.21 (d, *J* = 8.1 Hz, 1H, H-7), 6.92 (s, 1H, H-5), 6.91 (m, 1H, H-8), 6.82 (t, *J* = 7.5 Hz, 1H, H-9), 5.70 (s, 1H, H-5′), 5.18 (d, *J* = 16.9 Hz, 1H, H-10′), 4.38 (m, 1H, H-2), 3.81 (d, *J* = 3.8 Hz, 1H, H-2′), 3.73 (s, 3H, 1-OCH3), 3.49 (m, 1H, H-10′), 3.14 (dd, *J* = 14.6, 4.4 Hz, 1H, H-3), 2.27 (dd, *J* = 15.9, 3.8 Hz, 1H, H-3′), 0.67 (m, 1H, H-3′). ^{13}C NMR (176 MHz, MeOD) δ 168.7

(C, C-1′), 166.6 (C, C-1), 149.8 (C, C-6′), 147.8 (C, C-8′), 137.6 (C, C-7a), 135.1 (C, C-7′), 129.1 (C, C-10a), 129.0 (C, C-4′), 125.6 (CH, C-5), 122.5 (CH, C-8), 120.1(CH, C-10), 119.7 (CH, C-9), 112.0 (CH, C-7), 111.0 (C, C-9′), 109.1 (C, C-4), 107.4 (CH, C-5′), 60.9 (CH_3, 1-OCH_3), 57.7 (CH, C-2), 56.6 (CH, C-2′), 41.1 (CH_2, C-10′), 33.1 (CH_2, C-3′), 31.4 (CH_2, C-3).

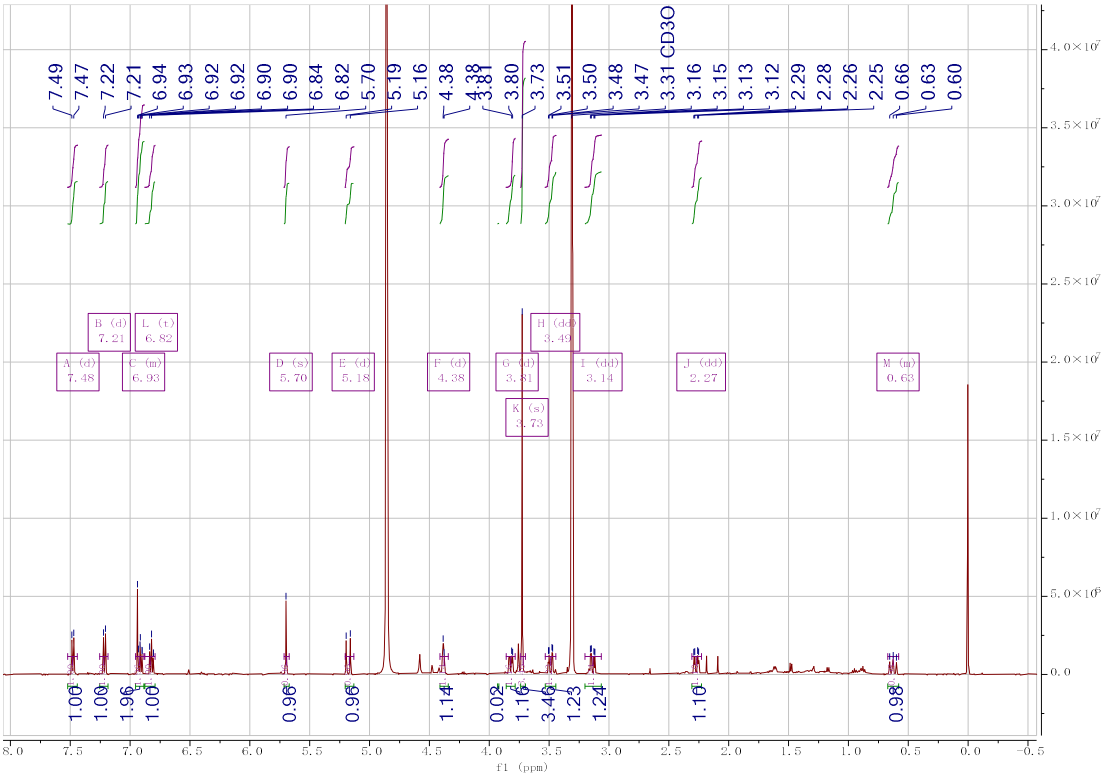

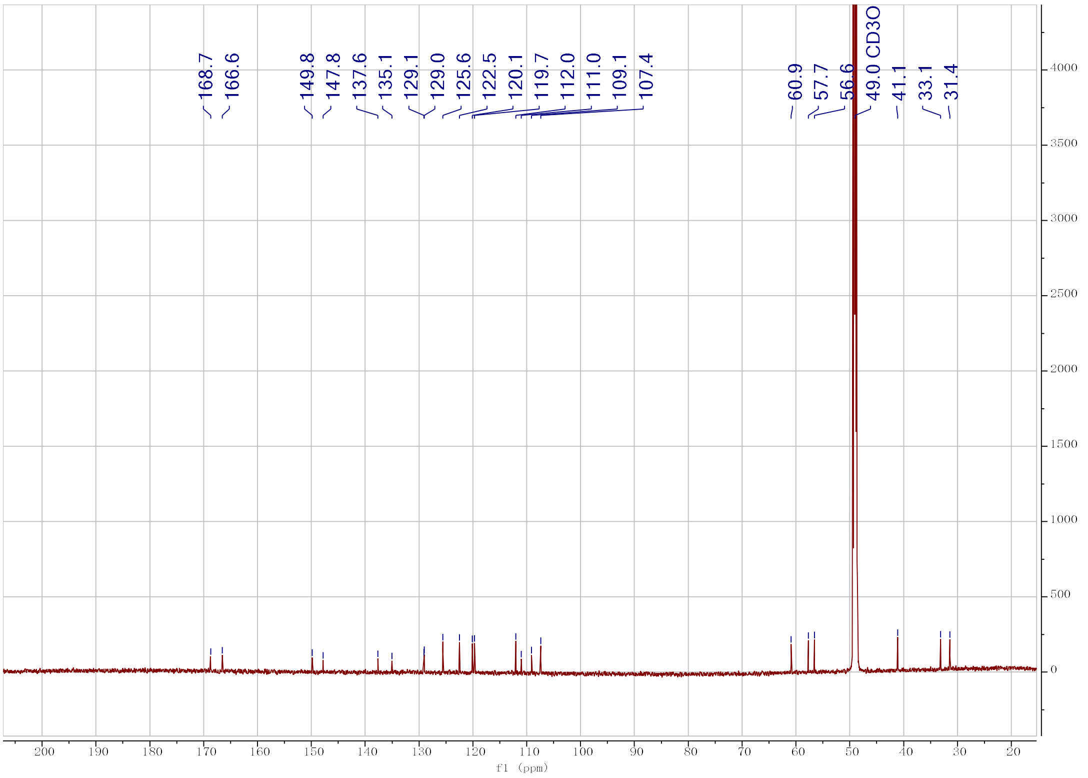

Supplement: Supplementary file 1 [file marinedrugs-21-00567-s001.zip › marinedrugs-2694454-supplementary.pdf]
